# Comparative Study of Fractionation Technologies and Their Impact on the Nutritional and Functional Properties of Maize and Rice Fractions

**DOI:** 10.1007/s11947-025-04104-0

**Published:** 2025-12-06

**Authors:** Ulrich Sukop, Viktoria Zettel, Carmen Boyaciyan, Regine Schönlecher, Katharina Höfler, Stefano D’Amico, Konrad J. Domig, Denisse Bender, Mario Jekle

**Affiliations:** 1 Department of Biotechnology and Food Science, Institute of Food Science, BOKU University, Muthgasse 18, Vienna, 1190 Austria; 2 Department of Plant-Based Foods, Institute of Food Science and Biotechnology, University of Hohenheim, Garbenstraße 25, Stuttgart, 70599 Germany; 3 Department of Biotechnology and Food Science, Institute of Food Technology, BOKU University, Muthgasse 18, Vienna, 1190 Austria; 4 Department for Feed Analysis and Quality Testing, Institute for Animal Nutrition and Feed, AGES – Austrian Agency for Health and Food Safety, Spargelfeldstraße 191, Vienna, 1220 Austria

**Keywords:** Cereals, Processing, Milling, Gluten-free, Nutrients, Efficiency

## Abstract

The two globally leading cereals, maize and rice, offer great potential for producing high-value fractions and biopolymers for diverse food and non-food applications. Wet- and dry-fractionation techniques are commonly used to concentrate specific kernel components, with process parameters adjustable to yield tailored functional fractions. This review provides a comprehensive analysis of the key biopolymers in maize and rice, their functional properties, and applications across sectors. It also examines both established and emerging fractionation methods, emphasizing how processing conditions influence nutritional and functional qualities. The interplay between kernel structure and process parameters is highlighted as a critical factor in optimizing yield and tailoring fraction properties. In wet-fractionation of maize, steeping conditions (time, temperature, acids, and SO_2_ concentration) and maize variety significantly affect protein and starch separation, fraction purity, and starch properties (pasting, gelling, and water solubility). Further, physical (e.g., ultrasonic and pulsed electric field) or enzymtic treatments have shown to increase wet-fractionation efficiency. In rice, dry-fractionation efficiency, especially of bran and head rice, is strongly affected by kernel shape and overall hardness as well as milling degree. Conversely, dry-fractionation in maize is typically used for particle size reduction, while wet-fractionation of rice is less common and often applied for soaking, solvent extraction milling, parboiling, or combined with dry grinding. Overall, the present study underlines the complex relationship between cereal structure, processing parameters, and final fraction properties. A deeper understanding of these interactions offers valuable insights into customizing processes for the efficient, targeted, and sustainable production of functional biopolymers and fractions from maize and rice.

**Supplementary Information:**

The online version contains supplementary material available at 10.1007/s11947-025-04104-0.

## Introduction

Apart from wheat, maize and rice constitute key sources of food for human consumption and industrial processing. Maize, for instance, represents a versatile, multipurpose crop that plays a crucial role in nutrition, contributing to more than 20% of the human caloric requirements. It is also predominantly used for animal feed and bioenergy production. The cultivation of maize is characterized by high adaptability and grain yielding ability across a broad range of environmental conditions (Ali, 2021). More than a half of the maize production, which is dominated by the USA and China, is used for feedstuff, while a fifth accounts for non-food uses (e.g., bioethanol) and processed or unprocessed food products (13%) (Erenstein et al., 2022).

Among the globally leading grain crops, rice covers about 21% of the world’s food calories and has an even stronger presence in Southeast Asia, accounting for up to 76% of the energy intake (Mohidem et al., 2022). Since maize and rice are inherently gluten-free (GF) cereals, they play a crucial role in both global food security and in the growing GF product market, which is mainly driven by medical necessity and consumer trends (Khairuddin & Lasekan, 2021). Rice processing by-products, including rice straw, husk, or bran, are currently employed in bioethanol production and as animal feed or are incinerated, resulting in a serious environmental issue. In order to overcome inefficient utilization, greater attention has been given to an extended application of extracted bioactive compounds from by-products in food and pharmaceutical industries over the past few years (Peanparkdee & Iwamoto, 2019).

Wet- and dry-fractionation of maize and rice kernels are commonly used to stabilize, remove, or extract specific grain components. Dry-fractionation mainly relies on the reduction of kernel or particle size by dry-milling, followed by the mechanical separation of the resulting fractions based on size, density, or shape (through, e.g., sieving and electrostatic methods), enabling targeted nutritional enrichment. In contrast, the water-intensive wet-fractionation technology facilitates a more efficient separation of the nutritional components (dietary fibers, starch, proteins, and lipid-rich germ) and involves alkaline or acidic steeping, wet-grinding procedures, boiling, or steam-cooking (Muchlisyiyah et al., 2023; Pulivarthi et al., 2023; Purewal et al., 2022). In literature about cereal processing, the term “milling” is often used with “fractionation” (Abdel-Aal, 2024). While “milling” generally implies particle size reduction, the term “fractionation” is preferred in this review to better emphasize the targeted separation and modification of kernel constituents, including relevant pre-treatments and subsequent steps after milling.

Given the uneven distribution of nutrients and bioactive compounds within the grain, both fractionation methods significantly affect the presence and concentration of the main biopolymers (dietary fiber, starch, lipids, and proteins) within the fractions, consequently determining their functional properties. These attributes are not only affected by the processing itself, but are strongly influenced by intrinsic factors such as growth conditions, grain variety, type of cultivar, and overall kernel properties. Consequently, by adjusting process parameters, these characteristics can be precisely controlled and optimized to obtain suitable fractions for a targeted applications in food products exhibiting a specific texture, density, flow behavior, appearance, as well as nutrient bio-accessibility, and absorption (Zhang et al., 2023).

Therefore, this review aims to comprehensively analyze the key biopolymers in maize and rice, highlighting their key attributes, functional properties, and applications across food as well as non-food sectors. It further seeks to assess both established and innovative fractionation methods, with their corresponding processing conditions, to identify strategies for tailoring nutritional and functional fraction characteristics. In addition, the review aims to underline the complex interplay between kernel properties and processing parameters, identifying critical thresholds for both aspects.

To support this analysis, relevant peer-reviewed articles and books were collected using Google Scholar and Scopus. The search strategy included selected keywords (and synonyms) and their combinations related to the main topics, e.g., maize/corn, protein, and maize protein fractionation. The aim is not only to summarize existing knowledge, but also to compare methodological approaches, highlighting the impact of processing conditions on fraction properties. In addition, it identifies critical knowledge gaps regarding the interaction between cereal structure and processing. These aspects are essential for guiding future research and for developing efficient, targeted, and sustainable fractionation strategies.

## Cereal Biopolymers: Distribution, Functional Properties, and Role in Maize and Rice

Maize and brown rice kernels possess distinct differences in their compositional profile, which are critical for designing effective fractionation strategies. Maize kernels are characterized by a relatively larger germ (∼11%), a pronounced tip cap, and a thicker bran layer, contributing to its relatively higher protein and fat content in the germ and bran. In contrast, brown rice possesses a higher proportion of starch particularly within the endosperm but generally contains less protein and fat than maize kernels. The structural and compositional differences within the kernels, in combination with the applied fractionation method, have a direct impact on the efficiency of recovery, as well as on the nutritional and functional quality of the resulting fractions. A detailed breakdown of the starch, protein, dietary fiber, and fat content across these cereals and their fractions can be taken from the Supplementary Table 1.

### Starch

Starch is the dominant biopolymer in both cereals and mainly located in the endosperm. Depending on the botanical origin, starch granules vary significantly in their characteristics, such as granule size (~ 1–100 µm), shape (round, lenticular, and polygonal), size distribution (uni- or bi-modal), arrangement (individual granules or in clusters), chemical composition and the ratio of the two main components amylose (linear polymer of 1,4-bound glucose units; molecular weight of ~ 10^5^–10^6^ Da) and -amylopectin (branched polymer with 1,4- and 1,6-bound glucose units; molecular weight of ~ 10^8^ Da) (Cornejo-Ramírez et al., 2018). Beyond genetic and structural characteristics, environmental factors also play a critical role in determining the physicochemical and functional properties of starch. For instance, high growth temperature (~ 35 °C) or water deficiency could significantly lower the amylose content (of about 20%), while water and salinity stress can alter starch pasting properties (e.g., rapid swelling and increase in peak viscosity) (Ronie & Mamat, 2022).

Starches are commonly classified according to amylose content as “waxy” (also referred to as “glutinous” or “sticky” in both maize and rice; amylose content of 0–8%), “normal” (20–40%), or “high amylose” (50–90%), although exact values can vary among cereal types and cultivars (Li et al., 2022). In maize, amylose content of normal starches is reported at 21–30% (Cornejo-Ramírez et al., 2018; Talukder et al., 2025; Wang et al., 2014, 2025), while waxy types contain around 0–5% (Talukder et al., 2025; Wang et al., 2025) and high-amylose types about 55–70% (Liang et al., 2025; Moreno-Zaragoza et al., 2025; Zhang et al., 2025). In rice, waxy types comparably contain 0–5% (Liu et al., 2021a, b; Luo et al., 2021), while normal types are slightly lower in amylose content, ranging between 13 and 27% (Cai et al., 2015; Liu et al., 2021a, b; Luo et al., 2021), and high-amylose rice starch was reported at > 33% (Luo et al., 2021; Singh et al., 2012). Maize starch granules (waxy and normal) have either spherical or polyhedral shapes and are unimodally distributed, and the granule size is 1–30 µm. Rice starch occurs only in the polyhedral shape, the granules are unimodally distributed, and their size ranges from 1 to 8 µm as single granule and up to 150 µm as naturally occurring aggregates in the rice endosperm (Cornejo-Ramírez et al., 2018). These granules comprise a complex structure of semi-crystalline and amorphous ring layers, in which the crystalline regions are composed of densely packed double helices formed by amylopectin side chains. Moreover, amylose chains bind with amylopectin molecules from different layers, predominantly in the outer regions. These interconnecting chains contribute to an increased stability of the starch granule, preventing it from extending during heat treatment (Zhiguang et al., 2025).

Due to its outstanding functional characteristics, starch is widely used in a wide range of industrial applications (e.g., thickening, gelling, or bulking agent). The thermal properties of starch including gelatinization and pasting are well described in literature and significantly affect the overall functionality of starch (Donmez et al., 2021; Schirmer et al., 2015). In this context, starch–water interactions represent key contibutors to the thermal stability and structural transition of starch during processing and storage. Moreover, its functional performance (e.g., gelatinization temperature and viscosity) is highly sensitive to variations in product formulations and processing parameters, including moisture content, temperature, shear, pressure, centrifugation parameters, and physical treatments (e.g., ultrasound). However, intrinsic factors strongly influence starch behavior. These include particle size (including the A-type and B-type morphology), shape, amylose and amylopectin content, molecular structure (i.e., molecular weight, chain length distribution, molecular conformation, and crystallinity types), and starch damage, which can be induced by processing conditions such as heat and mechanical stress during milling. For example, waxy maize starch tends to be more readily digestible, whereas high-amylose starches contribute to enhanced levels of resistant starch. Micronization of high-amylose maize starch has been shown to strongly reduce crystallinity and molecular weight, resulting in a lower gelatinization temperature, decreased apparent viscosity, and faster hydrolysis compared to untreated starch. Furthermore, moist-heat or high-pressure treatment can induce significant changes in the molecular structure of the starch granule, decreasing the relative crystallinity and consequently altering the gelatinization behavior (Lv et al., 2021; Zhiguang et al., 2025).

Depending on the intended application, starch functionality is also modified chemically or physically, e.g., by pre-gelatinization or use of different grinding methods (Mounir et al., 2024). For instance, ultra-centrifugal grinding has been shown to alter starch functionality, as the mechanical stress and associated heat exposure can shift gelatinization onset and affect flour hydration (Paulik et al., 2019).

An overview of starch occurrence in different types and grain-based fractions from maize and rice is provided in Table 1 and Supplementary Table 1. Moreover, it highlights the overall biopolymer composition of selected cereal fractions and the potential optimization of fractionation conditions, as further discussed in the “Fractionation Technologies for Maize and Rice” section.

**Table 1:** Proportions [%] of key biopolymers in different maize and rice types and fractions. Values are given according to the provided sources and reflect the original significant digits

Type, Fraction	Starch [%]		Protein [%]		Fiber-associated [%]		Lipids7 [%]	
Brown rice	66	(Amagliani et al., 2017)	7.1–8.3	(Amagliani et al., 2017)	Crude fiber: 0.6–1.0 Crude fiber of bran: 7.0–11	(Amagliani et al., 2017)	2.9–3.2	(Jingyi Wang et al., 2021)
Rice (milled)	78	(Amagliani et al., 2017)	6.3–7.1	(Amagliani et al., 2017)	Crude fiber: 0.2–0.5	(Amagliani et al., 2017)	0.3–0.5	(Amagliani et al., 2017)
	12.12–18.19^1^, 57.6–61.37^2^	(Hu et al., 2020)^3^	6.86–7.61	(Hu et al., 2020)				
Rice varieties (milled)	82.04 ± 0.10 (carbohydrates)	(Ronie et al., 2022)	8.97 ± 0.08	(Ronie et al., 2022)	Crude fiber: 0.21 ± 0.03	(Ronie et al., 2022)	0.16 ± 0.03	(Ronie et al., 2022)
	79.25 ± 1.19 (carbohydrates)		8.66 ± 0.08		Crude fiber: 0.87 ± 0.05		2.45 ± 0.08	
Rice (white)	76.70 ± 3.04	(Cao et al., 2009)	7.38 ± 1.61	(Cao et al., 2009)	Fiber: 0.06 ± 0.01	(Cao et al., 2009)	0.27 ± 0.03	(Cao et al., 2009)
	51.1–55.0 (carbohydrates)	(Esa et al., 2013)	11.2–14.4	(Esa et al., 2013)			0.41	(Jingyi Wang et al., 2021)
Rice bran	14	(Amagliani et al., 2017)	11–15	(Amagliani et al., 2017)	Dietary fiber: 20.5–33.3	(Y. Liu et al., 2021a, 2021b)	9.5–22.9	(Y. Liu et al., 2021a, 2021b)
					Cellulose: 15.8; Hemicellulose: 31.3; Lignin: 11.6	(Arzami et al., 2022)	15–22	(Sharif et al., 2014)
	90 (carbohydrates)	(Esa et al., 2013)	10.6–16.9	(Esa et al., 2013)	Dietary fiber: 20–30 (90% of which are IDF)	(G. Zhao et al., 2018)	15–20	(Amagliani et al., 2017)
			54.08; 58.92; 52.46	(Chandi & Sogi, 2007)	Arabinoxylans: 4.8–5.1 β-glucan: 0.04–0.21	(Arzami et al., 2022)	9.5–22.9	(Y. Liu et al., 2021a, 2021b)
	Native: 19.5 ± 2.1 Stabilized: 19.9 ± 2.0 Commercial-defatted: 20.2 ± 0.5	(Dang & Vasanthan, 2019)	Native: 17.7 ± 0.0 Stabilized: 17.8 ± 0.0 Commercial-defatted: 19.3 ± 0.3	(Dang & Vasanthan, 2019)	Total dietary fiber: Native: 37.8 ± 1.4 Stabilized: 44.0 ± 0.3 Commercial-defatted: 36.9 ± 0.9	(Dang & Vasanthan, 2019)	Native: 20.7 ± 0.3 Stabilized: 22.9 ± 0.1 Commercial-defatted: 5.5 ± 0.0	(Dang & Vasanthan, 2019)
Rice husk	22.4–35.3 (carbohydrates)	(Esa et al., 2013)	2.1–4.3	(Esa et al., 2013)	Cellulose: 28.7–35.6; Lignin: 15.4–20.0	(Arzami et al., 2022)	0.30–0.93	(Esa et al., 2013)
			2.08 ± 0.02	(Jaichakan et al., 2021)	Arabinoxylans: 12.63 ± 0.03	(Jaichakan et al., 2021)		
Common maize	73.83 ± 0.04 (carbohydrates)	(Abiose Sumbo & Victor, 2014)	9.80 ± 0.01	(Abiose Sumbo & Victor, 2014)	Crude fiber: 2.60 ± 0.02	(Abiose Sumbo & Victor, 2014)	Waxy: 5.91	(Jingyi Wang et al., 2021)
Maize (white)	79.91 ± 0.10	(David et al., 2022)	8.48 ± 1.18	(David et al., 2022)	Fiber: 1.86 ± 0.08	(David et al., 2022)	2.05 ± 0.04	(David et al., 2022)
Maize (red)	64.80	(Žilić et al., 2010)	10.35	(Žilić et al., 2010)	Cellulose: 2.27	(Žilić et al., 2010)	5.71	(Žilić et al., 2010)
Maize (yellow)	71.64		8.29		Cellulose: 1.42		5.21	
Maize cultivars^5^	62.39–68.45	(Mut et al., 2022)	10.12–13.24^5^	(Mut et al., 2022)	Acid and neutral detergent fiber: 3.65–5.22; 13.96–16.41^6^	(Mut et al., 2022)		
Quality protein maize	73.98 ± 0.04 (carbohydrates)	(Abiose Sumbo & Victor, 2014)	9.72 ± 0.12	(Abiose Sumbo & Victor, 2014)	Crude fiber: 2.05 ± 0.01	(Abiose Sumbo & Victor, 2014)	4.50 ± 0.06	(Abiose Sumbo & Victor, 2014)
Maize bran	64.7 ± 2.6	(Roye et al., 2020)	6.2 ± 0.2	(Roye et al., 2020)	Crude fiber: 7.8 ± 0.3; 8.2 ± 0.4	(Hussain et al., 2021)	13.2–19	(Rose et al., 2010)
			11.8 ± 0.7; 9.8 ± 0.4	(Hussain et al., 2021)				
	9–23	(R. Zhang et al., 2021)	10–13	(R. Zhang et al., 2021)	Arabinoxylans: 10.7	(Roye et al., 2020)		
			0.91	(Mendonça et al., 2000)			6.4 ± 0.2; 7.3 ± 0.4	(Hussain et al., 2021)
	29.92–56.97 (carbohydrates)	(Sousa et al., 2019)	0.08–12.39	(Sousa et al., 2019)	Fiber: 12.3	(R. Zhang et al., 2021)		
Others	Maize pericarp: 83.2 ± 3.2	(Yoshida et al., 2010)	Maize pericarp: 9.5 ± 0	(Yoshida et al., 2010)	Maize straw: arabinoxylans: 27.0–30.0	(Bastos et al., 2018)	Maize germ (wet-fractionated): 40–50% Maize germ (dry fractionated): 20–25%	(Jingyi Wang et al., 2021)

^1^Amylose

^2^Amylopectin

^3^Eight varieties of japonica rice

^4^Not determined

^5^Nineteen cultivars

^6^Two cultivars

^7^Equated with the term oil, fat, and crude fat

### Protein

Proteins represent key components of food matrices, contributing to their structural and functional behavior. These biopolymers, from both plant and animal sources, differ substantially in their chemical composition and physical characteristics. Protein functionality is strongly influenced by the amino acids, protein size, structural organization, and molecular forces involved, as well as the interactions with other food components and the applied processing steps (Yada, 2018). Functional properties of interest of maize and rice proteins include water holding capacity (WHC) (AACC International, 2010), solubility (Grossmann & McClements, 2023; Praseptiangga et al., 2021), emulsification, foaming capacity, and gelation (Wang et al., 2023).

Maize as well as rice proteins can be categorized according to the Osborne fractionation (Ma et al., 2025; Osborne, 1924). The classification defines four main protein fractions based on their solubility in various solvents: albumin (water-soluble), globulin (salt-soluble), glutelin (alkali/acid-soluble), and prolamin (alcohol-soluble). A comparison of rice and maize protein solubility according to Osbourne can be taken from Supplementary Table 2.

The major storage protein (located in the endosperm) of maize is prolamin (zein), while that of rice is glutelin (oryzenin). In maize, the predominant fraction represents α-zein (70–85%) with a molecular weight of 19 and 22 kDa. All zein fractions exhibit amphiphilic properties, containing both hydrophobic and hydrophilic amino acids, and lack the essential amino acids lysine, methionine, and tryptophan (Devi et al., 2024).

In rice, glutelin is composed of two main polypeptide subunits with a molecular weight of 30–40 kDa (α-subunit) and 19–23 kDa (β-subunit). Further, this protein fraction shows low water solubility (at pH 3–10) and specifically contains relatively high amounts of disulfide bridges. Rice proteins exhibit a balanced amino acid composition and a higher biological value compared to other cereals. In particular, they contain increased amounts of lysine (1.3–5.1%) compared to maize and are rich in sulfur-containing amino acids. Importantly, the amino acid composition and distribution are strongly dependent on the cultivar, the protein fractions in the kernel constituents, and further processing steps (Jayaprakash et al., 2022; Jiang et al., 2024).

Maize proteins, especially zein, have garnered significant attention due to their hydrophobic nature and ability to form cohesive and continuous networks under elevated temperatures (Bean et al., 2021). The formation of such viscoelastic networks is of particular interest, as they strongly influence the textural and functional properties of food products. These properties have made zein (including nanoparticles and fibers) a promising candidate for the development of biopolymer-based and ecofriendly food packaging materials, nano-carriers (Ozcalik & Tihminlioglu, 2013), edible films, and coatings (Tadele et al., 2025). Moreover, maize proteins have been applied as a non-dairy alternatives in cheese-analogues, exhibiting temperature-dependent textural properties and stretchability comparable to those of conventional cheese (Rashwan et al., 2025).

On the other hand, rice proteins are characterized by their low water solubility and limited emulsifying activity, which can be largely attributed to their amino acid composition and structural arrangement, including high amounts of disulfide bonds. Processing can, however, be used to enhance their functional properties. For example, heat treatments may induce partial protein degradation and exposure of hydrophobic groups, thereby enhancing oil binding capacity. Other treatments, including freeze-drying, alkaline treatment, and high temperatures, have shown to increase the foaming capacity and stability, likely due to formation of a cohesive protein films around air droplets during unfolding. Similarly, the emulsifying performance of rice endosperm protein concentrates can be significantly improved by the aforementioned treatments, enabling their use as natural plant-based emulsifier alternatives in food products (Jayaprakash et al., 2022; Qamar et al., 2020).

From a nutritional perspective, rice bran protein isolates, which are particularly rich in lysine, are considered hypoallergic compared to milk protein added to infant formulations or gluten-containing products such as baking products. They also show increased digestibility and amino acid availability, primarily attributable to their protein composition, along with neutral flavor and colorless appearance compared to other plant-based proteins (Jayaprakash et al., 2022).

### Dietary Fibers

Dietary fibers (DF) are a group of non-starch, complex carbohydrates, primarily found in the outer layers of cereals (bran and husks). Besides DF, the bran also contains smaller quantities of starch, lipids, proteins, and ash. Table 1 provides an overview of fiber-associated components in the whole kernel and constituents of rice and maize.

Both corn and rice dietary fibers are predominantly insoluble and consist mainly of cellulose, hemicellulose, and small amounts of lignin, oligosaccharides (Y. Liu et al., 2021a, b), and fiber-associated phenolic acids (Godoy et al., 2013). The hemicellulosic content in the bran comprises more than twice the content of cellulose and is structurally different than that found within the kernel (Arzami et al., 2022). For instance, it has been reported that maize heteroxylans from endosperm are marginally more branched than those from the pericarp, indicated by the increased arabinose/xylose ratio (Rose et al., 2010).

Compared to rice, maize bran is a rich source of hemicelluloses, such as arabinoxylans (AXs). Their physicochemical properties, especially solubility or water binding capacity, are affected by structural features (molecular size and weight, chain length, covalent linkages, and (non-carbohydrate) side groups) and can be enhanced or modified by further processing steps and pre-treatments, including mechanical (e.g., ultrasound, microwave, and extrusion), chemical (acidic and alkaline), or enzymatic methods (Arzami et al., 2022; Cai et al., 2021). In particular, highly-branched maize AXs, known as corn fiber gum (CFG), have attracted considerable attention in the food industry due to their gelling capacity (via oxidative cross-linking of ferulic acid moieites), antioxidative activity, and film-forming as well as emulsifying properties, although the latter two need further investigation (Cai et al., 2021).

Maize fiber has also been recognized as a promising starting material for the bioconversion of xylose into non-cariogenic, low-calorie food sweeteners (e.g., xylitol) (Rose et al., 2010). In addition, maize bran hemicelluloses offer potential as a substrate for bioethanol production (Yue et al., 2022).

Comparably, enzymatically modified low-molecular-weight rice AX have been reported to exhibit considerable immunological activity, e.g., by enhancing the maturation of dendritic cells. However, this functionality is highly dependent on structural characteristics, which remain insufficiently understood and require further research (Cai et al., 2021).

Insoluble dietary fiber (IDF) derived from rice has been shown to support physiological functions related to microflora, intestinal transit time, stool size, and bulk, yet caused undesirable sensory and textural properties when applied in foods. Enzyme-micronization treatment has shown to decrease the IDF content (4–11 fold), potentially reducing these adverse properties, but negatively affecting water and oil holding capacity (Wen et al., 2017). Comparably, using maize bran IDF as a baking ingredient also resulted in an unsatisfactory product appearance, including loaf volume reduction as well as a decrease in sensory scores (flavor, color, and texture) (Rose et al., 2010). In contrast, the application of soluble fiber significantly enhanced shelf-life of baking products, minimized shrinkage of bread, and counteracted negative sensory properties (Kurek & Wyrwisz, 2015).

Agro-industrial by-products, including maize bran and husk, were also identified as low-cost substrates for enzyme production (Mule et al., 2024) and as sources of hemicellulosic and cellulosic nanomaterials that can be incorporated into bio-composite films (Chen et al., 2023). Moreover, the addition of microwave- and ultrasonicated fiber-based rice nanoparticles in biodegradable starch films has been reported to modify their mechanical properties (e.g., higher tensile stress compared to non-fiber starch films) and increase the thermal stability and water permeability (due to hydrogen bonding between the polymers) (Louis et al., 2022).

Overall, dietary fibers from maize and rice exhibit diverse functional properties and offer potential for applications beyond conventional food uses. However, their full exploitation, particularly of by-products from cereal processing, still remains limited. Structural modifications may further expand their functional versatility, enabling novel applications in food, packaging, and biomedical sectors.

### Lipids

Non-polar cereal lipids are predominantly located in the germ and aleurone tissues. They are primarily composed of free fatty acids (FA), mono-, di-, and triglycerides. Minor amounts of lyso-polar lipids are arranged to the starchy endosperm, playing a crucial role in forming amylose–lipid complexes and protein interactions (Pareyt et al,. 2011). As a by-product of grain fractionation, cereal germs provide high levels of polyunsaturated FAs (PUFAs), essential amino acids, bioactive compounds, minerals, and phytochemicals. Despite the high nutritional value, this fraction is mostly used as animal feed or treated as waste. The chemical composition of cereal germs is greatly influenced by variety, growing environment, fertilization, season, and subsequent processing steps, which could promote the transfer of triacylglycerols from the germ to the final flour. For instance, it has been reported that high oil maize strains contained up to ~ 25% fat content, approximately twice the amount found in germs (Jingyi Wang et al., 2021). Table 1 displays a brief summary of lipid content in maize, rice, and their by-products.

Rice bran and germ oils have already been extensively studied for their nutritional composition and functional properties. They provide a well-balanced source of palmitic, oleic, and linoleic acid and minerals: non-saponifiable lipids (y-oryranol), tocopherols, tocotrienols, plant sterols, phenolic compounds (Punia et al., 2021), iron (~ 60 mg/kg), magnesium (~ 3000 mg/kg), and low levels of sodium (2 mg/kg) (Rondanelli et al., 2021). Additionally, oil extracted from rice bran offers health-promoting activities (e.g., antioxidative or lowering low-density lipoprotein cholesterol) mainly attributed to its high amounts of γ-oryzanols and tocopherls. This oil shows a high thermal stability (high burning point) and is characterized by a hypoallergenic profile. It therefore serves as a good fat substitute in baking goods, milk- and meat-based formulations, and zero or low trans-fatty products (Punia et al., 2021).

Comparably, germ- and fiber-derived maize oil shows a high content of the linoleic and oleic acid and provides a source of vitamin E, provitamin A as well as phenolic acids, carotinoids (zeaxanthin and lutein), and phytosterols (Barrera-Arellano et al., 2019). In terms of food applications, maize oil has been studied as a flavor-supporting additive in sourdough bread. The lactic acid bacteria fermentation and the application of this oil has shown to increase the concentration of aldehydes, ketones, and furans. Moreover, breads with this oil significantly increased product quality and sensory acceptance (Wu et al., 2022).

On the other hand, promising non-food approaches have targeted germ-extracted maize oil as an alternative biofuel in diesel engines. It has been reported that a ternary biofuel system combining maize oil, low-carbon alcohols, and co-solvents (e.g., n-butanol and tetrahydrofuran) enables stable miscibility. In addition, specific genes in the maize genome (ZmFAD2-1) have been identified as potential regulators of linoleic acid biosynthesis (Zhang et al., 2020), offering opportunities to optimize maize oil composition for biofuel production. Similarly, rice bran oil has also shown promising application in biodegradable lubricant formulations as a compatible polymer additive in polylactic acid and polyvinyl materials or as nanoemulsion-based ingredient in cosmetics for UV, disease, allergy protection (Manoj Kumar et al., 2021), or as biodiesel alternative (Punia et al., 2021).

In summary, during cereal milling, valuable by-products such as germ and bran are often removed and used as animal feed. However, oils extracted from those maize and rice by-products show great potential for use as precursors in the development of functional food products, cost-efficient biofuel sources, in additives of cosmetics or in medicine.

## Fractionation Technologies for Maize and Rice

Efficient fractionation techniques are essential for isolating and concentrating valuable components from cereal grains, significantly contributing to the nutritional and functional properties of the resulting fractions. This is performed through a series of pre-treatment and separation steps, such as defatting, conditioning to loosen the kernel structure, polishing for bran removal, and milling for size reduction, followed by particle separation techniques. Depending on the amount of water applied, two main fractionation technologies are employed for processing cereals: dry- and wet-fractionation (Pulivarthi et al., 2023).

The following subtopics will highlight the application of dry- and wet-fractionation technologies of maize and rice, emphasizing the effects of kernel properties and processing parameters on the produced fractions. An overview of both fractionation processes is schematically illustrated in literature by Purewal et al. (2022) and Lisboa et al. (2025).

### Dry-Fractionation

The overall goal of cereal dry-fractionation involves the systematic and relatively gentle reduction in kernel size and the mechanical separation of the distinct fractions (bran, endosperm, and germ) by polishing or sieving, air classification, and electrostatic methods after milling. In contrast, the water- and energy-intensive wet-fractionation is employed to isolate the grain’s components (fiber, starch, protein, and germ), specifically aiming to separate starch from proteins by solubilization of the latter (Pulivarthi et al., 2023). The process of dry-fractionation is highly dependent on the type of cereal, intrinsic grain properties, and the intended purposes, including either the removal of the bran and/or germ or the production of specific coarse fractions and flours. In order to achieve products of the desired nutritional composition and functional properties, precise adjustment of processing conditions is required. Likewise, dry-fractionation efficiency is strongly influenced by intrinsic kernel characteristics. These complex interdependencies of maize and rice kernels and the applied methods will be further examined in the following sections “Relationship of Maize Kernel Properties and Dry-Fractionation Conditions” and “Effects of Dry-Fractionation Conditions on Rice Products”.

#### Relationship of Maize Kernel Properties and Dry-Fractionation Conditions

Prior to dry-fractionation, several preparatory operations must be carried out. First, the maize kernels, meeting quality criteria such as large and/or uniform kernel size and shape, test weight, moisture, high vitreous endosperm content, and absence of impurities are subjected to cleaning by passing through a magnet, aspiration, and sieving. Subsequent tempering is applied at constant moisture conditions to facilitate segregation of the pericarp, the aleurone layer, the endosperm, and the germ. This tempering procedure is applied either in multiple stages (usually 3, with residence time of 145 min) to achieve a final moisture content of 24% or in a single step, adding 6–8% moisture. After removal of the germ using various types of degerminators (ball, impact, and roller milling), the obtained fractions undergo a further drying step (to 15–16% moisture). This is followed by a series of milling processes to reduce the particle size of the grain, which is then separated by means of sieving into different fractions. Dry fractions of maize can generally be classified into five categories including grits, meal, flour, germ, and hominy feed. This determination is based on the particle size, nutritional composition, and quality parameters, influencing further distribution and application (Purewal et al., 2022).

The hardness of the maize kernel is a key determinant of the dry milling performance and therefore an important grain quality attribute (Blandino et al., 2013). It is predominantly influenced by the pericarp thickness and ratio of floury and vitreous endosperm, which depends on maize variety (e.g., flint, dent, and floury maize), as well as starch and protein composition; the latter is, in turn, affected by the soil nitrogen level.

In addition, moisture content represents another critical physical parameter, as it influences various other characteristics including density, weight, and elasticity (Purewal et al., 2022). The final moisture of the kernel before at harvest is governed by its initial level at physiological maturity, which is primarily genetically controlled, and by the dry-down rate of the field, driven by temperature and relative humidity (Li et al., 2021). These maize kernel’s properties in turn affect the milling efficiency and capacity or feed rate (Purewal et al., 2022), energy consumption and remaining costs, milling type and breaking behavior, particle size as well as nutritional composition, and digestibility of the ground material (Lyu et al., 2022).

Table 2 displays an overview of the selected key kernel properties that affect processing parameters and emphasizes the interdependencies between the applied conditions (with a particular focus on milling) and the resulting physical, nutritional, and functional characteristics of the final fractions. This overview highlights the critical need of precisely adjusting dry-fractionation parameters to preserve or target functional properties.

**Table 2:** Relationship of maize kernel properties and applied dry-fractionation conditions, affecting process efficiency and product characteristics

Kernel properties/fractionation parameters	Fractionation efficiency/effects on product properties	Source
A. Hardness (affected by endosperm and cell wall structure) B. Density C. Weight/size (strongly correlating with A. and B.) D. Moisture E. Starch granules integrity (affected by microbial kernel quality, enzymatic activity, drying, storage, milling)	A. Bulk density, milling resistance/power, grinding resistance, breakage susceptibility, dust formation, fraction yields B. Bulk density (test weight), porosity C. 1000-kernel weight, size D. Product water content, shrinkage E. Pasting properties	(Purewal et al., 2022)
Kernel hardness (↑)	• Coarse (↑^1^)/fine (↓^2^) ratio • Total milling energy (↑)	(Blandino et al., 2013)
Kernel density, total milling energy, test weight	• Product yield	(Blandino et al., 2017)
Moisture (↑)	• Handling costs (↑) (additional drying step required) • Microbial load	(Purewal et al., 2022)
Moisture (prior hammer milling) (↑)	• Kernel plasticity and energy consumption (↑) • Coarse particle yield (↑) • Geometric mean diameter (↑)	(Lyu et al., 2022)
1. Moisture of kernels: A. 11.1%, B. 17.6% 2. Hammer type: A. Less cutting shapes, B. More cutting shapes	• Application of 1. A. + 2. B./1. B. + 2. A.: Energy consumption (↓) • Application of 1. A. + 2. A./1. B. + 2. B.: Feed rate [kg/h] (↑)	(BUDĂCAN et al., 2013)
Protein content	• Product yield (positively correlating) • Influence on kernel shape and density	(Blandino et al., 2013)
Particle size (Fractions [mm sieve opening]: F1: 3.360, F2: 2.380, F3: 1.680, F4: 0.841, F5: 0.420)	• F1, F4, F5: (↑) Protein content • F4, F5: (↑) Neutral detergent fiber content	(Lyu et al., 2022)
Particle size (Fractions [mm]: F1: 0.125; F2: 0.250; F3: 0.5; F4: 1.0; F5: 2.0)	• Dominating nutrient in fractions: o Fiber content in F5 o Moisture in F4 o Protein content in F3 o Ash content in F2 • Functional properties of fractions with increasing particle size: o Water solubility (↓) o Water absorption (↓) o Oil absorption (↑) o Emulsifying capacity (↓) and stability (↑) of F5 compared to F1–4	(Sousa et al., 2019)
1. Maize types: A. Conventional hybrid, B. High-amylose hybrid, C. Waxy hybrid 2. Fraction types: Flour (< 150 µm), break meal (250–500 µm)	• Nutritional compositions of fractions: Fat (highest in flour b.), fiber and protein (highest in break meal b.), damaged starch content (lowest in break meal a.) • Pasting properties	(Bresciani et al., 2021)
1. Maize types: A. White dent, B. Yellow popcorn, C. Red dent, D. Blue popcorn 2. Particle size (1700 μm, 710 μm, 212 μm)	• Highest overall in vitro digestibility and lowest starch content (50.8%) of yellow popcorn fraction (highest: < 21 µm) • Highest crude fiber content in fractions of particle size 1700–710 µm • (↑) Particle size → (↑) protein content	(Nikolić et al., 2023)
1. Maize type: A. Flour, B. Semolina of yellow maize, C. Flour of white maize 2. Particle size	• Color: higher color values (a* and b*) in yellow maize; lighter and less yellowish color in fine particles • Dough and bread properties: o (↓) of G″/G′ while particle size (↑) (C.) o Finer flours → lower dough development during fermentation o Coarser flours → (↑) bread volume, less firmness	(La Hera et al., 2013)
Milling fraction types	Total antioxidant capacity, phenolics, fiber, and xanthophyll content	(Blandino et al., 2017)
Mill types: A. Hammer, B. Roller mills	A.: Easy but cost-intensive, less uniform particle size production B.: Precise, controlled, less dust producing	(Hafeez et al., 2015)
Tip speed of hammer mill (↑)	• Electrical energy consumption, coarse grit particle size (↓) • Capacity of machine, milling efficiency, economic viability (↑)	(Dominguez, 2021)
Milling speed (mini flour stone mill: 75, 115 rpm; commercial flour mill: 400 rpm)	(↑) Milling speed affected: • Milling time, flour recovery (for sieve size of 1.4 mm) (↓) • Flour temperature, feed rate (↑) • Nutritional storage stability (evaluated through nutrients) (↓)	(Sidhu et al., 2016)
Type of degerminator: A. Beall B. Impact C. Multiple impact/shear D. Roller milling	Fraction yields, separation efficiency: A. Large flaking grits, effective germ removal B. Reduced germ and pericarp separation efficiency C. Less yield of flaking grits D. No recovery of flaking grits, less effective germ, and pericarp separation	(Purewal et al., 2022)

^1^Positive effect/increase

^2^Negative effect/decrease

#### Effects of Dry-Fractionation Conditions on Rice Products

Similar to maize, rice processing starts with an initial cleaning of the raw rice kernels followed by the actual dry-fractionation steps. These include the dehusking of the paddy rice, the dehulling of brown rice to obtain polished rice, the separation of broken and unbroken rice (grading) resulting in head rice, and the final blending procedure (Singh et al., 2018).

Especially, the removal of the bran and outer layers, also called polishing, pearling, or whitening, represents the most crucial procedure in rice dry-fractionation, which is notably distinguished from that of maize, primarily aiming at particle size reduction. In this regard, the degree of milling (DOM) is a crucial parameter in rice processing, which is commonly calculated either as the percentage of bran removed from brown rice (Kalpanadevi et al., 2018), or as the ratio between the weights of polished and brown rice (Liu et al., 2022; Shin et al., 2022). The gradual separation of the outer layers is predominantly determined by the milling time and affects both the nutritional and functional characteristics of the final product (compare Table 3). The amount of bran which is removed at a certain time depends on the rice variety and kernel properties such as its shape (length and aspect ratio) and the hardness of the different bran layers as well as of the outer endosperm fraction. This polishing process typically employs either abrasive or friction-type polishing machines. In friction-type polishers, rice kernels rub against each other, generating pressure that detaches the bran. In contrast, abrasive-type polishers remove the bran at high speed through friction between the rice kernels and the polisher roll.

**Table 3:** Effect of dry-fractionation conditions on process efficiency and rice product properties

Fractionation parameters	Fraction	Effects on process/product properties	Source
Tempering, drying prior fractionation	Unparboiled brown rice	• Relative humidity, temperature, air flow, and solar radiation on drying performance • (↑)^1^ Drying time and temperature (↓)^2^ moisture, (↑) kernel hardness, (↓) kernel breakage • Highest milling yield: 5 h sun drying; highest head rice recovery: 4 h drying	(Keya et al., 2025)
Degree of milling (DOM) (↑)	Rice varieties	• (↑) of brightness/L* color value • Thermal properties: Gelatinization temperature (↓) and energy ΔH (↑), degree of gelatinization (↑) • (↓) Gel consistency (negatively correlating with amylose content) when (↑) of DOM • Protein (↓), ash (↓), total starch (↑), total and insoluble (↓) and soluble dietary fiber (→)^3^ • Significant differences among varieties	(S. Zhao et al., 2023)
	Polished rice	• (↓) Ash, fat, dietary fiber, protein, vitamin B1 and B2	(J. Liu et al., 2022)
		• Protein, fiber and lipids (↓), starch (↑) • Starch damage (↑) • Hardness and adhesiveness (↓) of cooked rice	(Zhang et al., 2023)
		• Fat, protein (e.g., threonine) and ash (↓) • Riboflavin, thiamine, phytic acid (↓) • (↑) bioavailabilty of endosperm-related minerals (Cu, Ca, Zn, Se)	(Liu et al., 2017)
		• (↑) Glycemic index values, starch digestibility	(Farooq & Yu, 2025)
	Rice bran	• Ash, fat, proteins and essential amino acids (↑) • Secondary protein structure: (↑) β sheets, random coils, α-helix, and β-turns • (↓) Emulsifying stability index (increased relatively at higher pH) • Significant effect of bran-defatting on nutrients and functionality	(Kaur et al., 2021)
		• Albumin (↑), globulin, glutelin, prolamin (** →**) • Protein fractions and amino acid composition (** →**)	(Shin et al., 2022)
	Bran of black/red rice	Protein (↓), ash (↓), fat (↓), flavonoids (↓), bound and free phenolics (↓), antioxidant activity (↓)	(Paiva et al., 2014)
1. Roller mill 2. Hammer mill 3. Pin mill A. Pre-freeze grinding step (otherwise without)	Milled polished rice	• Sieving yield (correlating with milling efficiency): 1.: highest (91%), 3.: lowest (39%) • Average particle size: (↑) applying 1., (↓) with 3., lowest using 2. A • Damaged starch: (↓) applying 1./2. A • (↑) Structural breakage angles of starch granules with A	(Ngamnikom & Songsermpong, 2011)
1. Milling speed 2. Milling duration	Rice types: a.: waxy, b.: low amylose, c.: high-amylose	• (↑) of 1. + 2.: Protein and lipids (↓), total starch (↑), amylose content (→) • (↑) of 2.: Swelling power (↑), water solubility of c. (↓) • (↑) of 1. + 2.: Peak and breakdown viscosity (↑) • (↑) of 1. + 2.: (↓) Gelatinization temperatures (except c.); 2. greater influence on thermal properties than 1 • 2. Affects starch-digestibility of a.; 1. affects starch digestibility of b. and c	(Qiu et al., 2019)
One- and two-step air classification (for protein enrichment)	Milled rice bran fractions	• (↑) Separation efficiency applying two-step air classification • (↑) Protein solubility (at pH 5/7) of air-classified fractions compared to raw material; enhanced when applying two steps • Colloidal stability for fine fractions compared to raw material • (↓) Carbohydrates, starch, insoluble dietary fiber; (↑) phytic acid and ash in fine fractions	(Silventoinen et al., 2019)
Air classification after: 1. Single sieving 2. Pin milling and sieving 3. Double sieving	Rice bran fractions	• 1. + 2. + 3.: (↓) coarse and (↑) fine particle yield with (↑) of air flow rate • 1.: Protein (↑) in fine fractions • 2.: Ash (↑) in fine particles with (↑) of air flow rate • Soluble fiber (↑) applying 2	(Jayadeep et al., 2009)
Additional electrostatic separation after milling		• (↑) Separation efficiency of fiber and starch • (↑) Swelling capacity, water retention capacity, oil holding capacity compared to original bran	(Jue Wang et al., 2016)
Post dry-milling flour refining	Rice dust, colored rice	• (↑) Aggregation of starch granules, (↑) proteins, phenolics in rice dust • (↑) Pasting properties in rice dust, (↓) in colored rice • (↑) Antioxidant, UV-protective functionalities of colored rice • (↑) Mechanical properties of bio-based plastics from rice dust compared to refined rice starch	(Brites et al., 2025)

^1^Positive effect/increase

^2^Negative effect/decrease

^3^No significant effect

Additional milling steps (using, e.g., stone, colloid, hammer, pin, cyclone, hammer, and jet mill) may be required for the production of rice flour or rice starch from polished (or brown) rice (Bala, 2020). Subsequent fractionation of these dry-milled rice flours involves, for instance, energy-efficient air classification, which separates protein- and starch-rich fractions based on their densities (Tabtabaei et al., 2023) and/or sieving to adjust fiber and protein content based on flour particle size (Silventoinen et al., 2019; Jue Wang et al., 2016).

In addition to altering the nutritional distribution, dry-fractionation can yield fractions with enhanced functional properties. For instance, rice bran concentrates exhibited higher protein solubility (~ 8–26% soluble proteins at pH 4–7) compared to brown (6–20%) and white rice (2–23%) during subsequent extraction, which was attributed to differences in protein fraction composition and molecular weight. Higher protein solubility was positively correlated with improved emulsifying and foaming properties. Moreover, rice bran proteins with a high protein content and surface hydrophobicity can exhibit increased oil absorption capacity in comparison to whey protein (up to + 12%), casein, or ovalbumin. These functional properties underline the potential application of rice bran proteins as versatile food ingredients: they can act to reduce interfacial tension in foams (beneficial in sponge cakes and whipped creams), enhance flavor retention, and improve mouthfeel in meat analogues (Al-Doury et al., 2018; Kumar et al., 2024; M. Zhao et al., 2020).

Table 3 provides an overview of selected rice dry-fractionation parameters, focusing on polishing and subsequent milling conditions as well as classification steps. It highlights their influence on key functional and nutritional properties of the resulting fractions as well as the relevance of process parameters on desired product characteristics.

### Wet-Fractionation

Wet-fractionation technology is mostly applied in maize processing, particularly in the field of starch production, and yields high-value by-products enriched in fiber, protein, or lipids. In contrast, rice is less commonly subjected to wet-fractionation (Rosentrater & Evers, 2018); however, par-boiling plays a crucial role in modifying the structural and functional kernel properties (Serna-Saldivar, 2019).

As described in the previous subtopics, dry-fractionation primarily targets the physical size reduction of the whole kernels (or pre-milled fractions) and the subsequent separation of particles based on size, density, and/or shape. In contrast, wet-fractionation enables a more efficient separation of kernel constituents, yielding fractions of high purity. This is mainly facilitated by kernel hydration and chemical modifications during steeping, which degrades the protein matrix and promotes the release of starch granules (Sayaslan et al., 2023).

The following sections “Impact of Wet-Fractionation Conditions on Maize Properties” and “Wet-Fractionation Strategies in Rice Processing” provide a comparative overview of the key wet-fractionation processes, strategies, and conditions applied to maize and rice, highlighting their impact on the composition and functionality of the resulting fraction, with Table 4 providing a summary of the main findings.

**Table 4:** Effect of wet-fractionation strategies and conditions on process efficiency and maize as well as rice fraction properties

Method, procedure	Conditions	Cereal, fraction	Effects on fraction	Source
Wet-fractionation, steeping	0.1–0.2% SO_2_	Maize (whole kernels)	• (↓)^1^ Microbial growth • Breakage of disulfide bonds in protein matrix (↑)^2^ solubilization	(Delcour & Hoseney 2010)
	Lactic acid addition		• (↑) Protein-starch separation efficiency	
	pH 3.0, 3.5, 4.0, 5.9 (citric acid, 50 °C)		• (↑) Protein solubilization at pH 3, 3.5 (within 20 h)	(Biss & Cogan, 1996)
	SO_2_ (2500 ppm) with/without 0.5% (v/v) lactic acid (52 °C, 40 h))		• Lactic acid addition (↑) starch yield over time • Lactic acid addition (↑) protein yield (slight decrease over time) • (↑) Steeping time (↑) temperature, gelatinization enthalpy of starch ((↓) with lactic acid addition)	(Pérez et al., 2001)
	SO_2_ + enzyme addition (fiber-, protein-degrading)		• (↓) Steeping time (< 6 h), water consumption (↓) costs compared to SO_2_ only • (↑) Starch, protein yield	(Singh & Johnston, 2004)
	SO_2_ source, acids, initial pH 3	Maize (varieties)	• Starch yield affected by SO_2_ source, steeping acid, maize variety • Application of lactic, acetic acid, Na_2_S_2_0_5_ (↑) starch yield • Highest peak, final viscosity of starch using Na_2_S_2_0_5_, but hybrid-dependent	(Yang et al., 2005)
	SO_2_ presence	Maize gluten meal	• Distinct protein distribution in fractions functionality • Gluten meal (originated from endosperm): rich in zeins, glutelins	
	SO_2_ (0.1 and 0.2%), time (4, 6, 8, and 10 h)	Maize grits	• (↓) Kernel size (↓) required steeping time, SO_2_ concentration; (↓) starch viscosity	(Eckhoff et al., 1993)
	SO_2_ (0.05, 0.30%), lactic acid (0.2, 1.5%), steeping temperature (43, 57 °C)	Starch-rich fraction	• (↑) Temperature (↓) pasting, shear-thinning viscosity • (↑) Temperature Starch granule annealing; (↑) effect of lactic acid on reducing pasting, shear-thinning viscosity • (↑) Temperature, lactic acid, SO_2_ concentration water solubility • Intensive conditions (57 °C, 1.5% lactic acid, 0.3% SO_2_) less smooth, round starch granule surface	(Shandera & Jackson, 1996)
Wet-fractionation	PEF-treatment	Steeped, milled maize kernels	• (↑) Fiber, fat, protein solubility from fibers • (↑) Bio accessibility of phenolics	(Sukop et al., 2025)
	Ultrasound-application	Mill starch	• (↑) Starch yield (15 min, 200 W) • (↓) Steeping time, water, SO_2_ • (↑) Peak viscosity of starch, (↓) yellowness	(Liu et al., 2020)
Soaking and wet-milling	Soaking time (7 h, 7 d), temperature (25 °C, 5 °C)	White rice kernels	• (↓) Starch damage, (↑) leaching of fat, proteins compared to dry-grinding • (↑) Soaking time (↓) flour particle size, (↑) setback, breakdown, final viscosity, (↓) pasting temperature	(Chiang & Yeh, 2002; Yu et al., 2024)
Wet-milling	Wet-milling, centrifugation		• (↓) Starch damage, (↑) structural integrity, gelatinization enthalpy compared to dry-milled starch • (↓) Bioactive compound (gamma-aminobutyric acid)	(Zhang et al., 2025)
Semi-wet-fractionation	Soaking, drying, grinding	Brown rice	• (↓) Mechanical, thermal stress preservation of biopolymer functionality • (↓) Starch damage compared to dry-fractionation • (↑) Average starch granule size decline in pasting, thermal properties	(Asmeda et al., 2016)
Parboiling	Conditioning (30–35% moisture), heating, drying	Paddy rice	• (↓) Kernel hardness (↓) breakage, (↑) head rice yield • (↑) Storage, cooking stability • (↑) Nutrients: lipids, minerals, vitamin B; digestibility • Energy- and cost-intensive	(Muchlisyiyah et al., 2023; Serna-Saldivar, 2012)
Solvent extraction milling	Soaking, wet-milling (with hexane)	Brown rice	• (↑) Separation efficiency of bran, oil, head rice • (↓) Storage ability of bran, head rice • (↓) Oil quality ((↑) glycolipids, phospholipids, waxes, free fatty acids) • (↑) Environmental impact	(Kaimal et al., 2002; Rosentrater & Evers, 2018)

^1^Negative effect/decrease

^2^Positive effect/increase

#### Impact of Wet-Fractionation Conditions on Maize Properties

The wet-fractionation process is centered around the initial steeping stage, in which kernels are soaked for 24–40 h at 50 °C, followed by a series of milling and separation steps, including sieving, centrifugation, and sedimentation. This procedure is particularly important for facilitating the separation of the single constituents.

Sulfur dioxide (0.1–0.2%) is added as an essential part of the steeping water, not only to release starch from the protein matrix but also to inhibit the growth of putrefactive microorganisms. Within the steeping phase, bisulfite ions react with the disulfide bonds of the protein causing a reduction in the molecular weight of proteins, thereby increasing their solubility. The addition of lactic acid softens the grain and lowers the pH, thus aiding protein degradation (Delcour & Hoseney, 2010). It has been suggested that steeping efficiency, and more specifically the effect of SO_2_, can be improved under acidic conditions. Decreasing the pH value of the steepwater (from 5 to 3) significantly increased the impact of SO_2_ on the maize kernels, the solubilization of the kernels insoluble proteins, and their subsequent release from the starch granules, particularly within 20 h of steeping (Biss & Cogan, 1996). Further, it has been shown that the addition of sulfur dioxide significantly influenced both the distribution of the solubilized proteins in the resulting fractions as well as their functionality. In particular, compared to proteins in the endosperm, produced wet maize gluten exhibited a lower content of albumins, globulins (~ 36%), γ-zeins, and glutelin-like proteins (~ 26%) due to partial elimination of salt-soluble proteins during the isolation process (Landry et al., 1999).

Additionally, other wet-fractionation parameters such as temperature or the concentration of lactic acid could modify the functional properties of starch. Pasting and shear-thinning viscosities were reduced to greater extent by steeping at 57 °C compared to 43 °C. In this respect, higher concentrations of lactic acid (1.5% instead of 0.2%) or SO_2_ were less likely to affect pasting properties than the applied temperature and there probably was no additional impact of residual protein content (< 0.5%). In contrast, gelling (set-back) viscosity was more notably influenced by lactic acid concentration than steeping temperature. These steeping-induced alterations in starch functionality may be attributed to structural modifications, particularly the partial depolymerization of amylopectin. Moreover, it was shown that the water solubility of starch was increased by maize steeping levels of temperature, lactic acid concentration, and their interaction. Starch granules obtained from intensively steeped maize (57 °C, 1.5% lactic acid, 0.3% SO_2_) possessed more ridge-like, less round, and smooth surfaces compared to less intensively steeped maize starch (43 °C, 0.2% lactic acid, 0.3% SO_2_); however, there was a great variability in size and shape (Shandera & Jackson, 1996).

Further investigations regarding starch pasting properties and yield have shown that latter can be affected by using different SO_2_ sources (sodium metabisulfite, potassium metabisulfite, potassium sulfite, and ammonium sulfite) and steep acids taking into account acid normality, pH value, and maize variety. Weak acids such as lactic and acetic acids led to higher starch yields compared to stronger acids including hydrochloric, sulfuric, phosphoric, or oxalic acid at the same pH value. Furthermore, slight differences in the pasting properties of starch may occur when using various sulfite salts and acids, with no discernible trend (Yang et al., 2005).

Lactic acid has shown to enhance starch yield compared to steeping with SO_2_ alone. It was observed that lactic acid stabilized the thermal properties of starch during steeping, making it less sensitive to changes induced by annealing and steeping time. Further, enhancing the steeping time resulted in a greater release of solids, such as proteins, into the steeping liquor. By applying lactic acid as additional treatment, enhanced proteolytic activity can occur, subsequently leading to increased leaching of solids, due to a higher nutrient solubility (Pérez et al., 2001).

During fractionation, various strategies have been explored to enhance starch yield as well as the separation efficiency of the kernel’s constituents. Studies indicate that starch retention rates are largely determined by the location of starch granules within the kernel. Particularly in the fiber fraction, the limited starch retention (up to 10%) could be attributed to polysaccharide- or protein-related cell wall cross-links. Improved starch and protein separation from wet-milled maize fiber fractions can be achieved by using additional milling passes, application of enzymes (cellulases, xylanases, proteases, hemicellulases), chemicals (buffers containing sodium bisulfite, lactic acid, and acetic acid at pH 4), or physical treatments (ultrasonic homogenizer and pulsed electric field treatment (PEF)). In particular, the combination of enzymes had a significant effect on disrupting the dense protein and non-starch polysaccharide matrix of the endosperm’s peripheral part, as well as altering the secondary structures of proteins and molecular conformation of starch (Ozturk et al., 2021).

The use of proteolytic enzymes has shown to significantly increase starch yields (26.1% compared to conventional milling) as well as facilitate the separation of starch and proteins from maize fiber, even with reduced amounts of SO_2_ (600 ppm) and shorter steeping times (12 h) (Somavat et al., 2021). An ultrasound-assisted lab-scale wet-fractionation has been shown to achieve a 10% increase in starch yield. This can be accomplished by exposing a slurry with a liquid–solid ratio of 1:1 to an ultrasonic power of 200 W for 15 min. The starch isolated using ultrasound technology exhibited no significant differences in granular and crystalline structure as well as similar thermal properties (measuring the range and enthalpy of gelatinization) compared to the starch obtained from traditional wet milling. However, lower yellowness, higher peak, trough, breakdown, and final viscosity and larger storage moduli (G′) were observed (Liu et al., 2020).

Moreover, PEF has been used as pre-treatment step during wet-fractionation to enhance fractionation efficiency, while only inducing minor structural changes in the fraction’s biopolymers. It altered the nutritional composition of the fractions by enhancing fiber, fat, and protein solubility. The fiber-rich fractions also showed higher water-holding capacity and greater accessibility of phenolic side groups, reflected in increased total phenolic content. In the protein-rich fraction, PEF led to a clear reduction in surface hydrophobicity, likely due to the exposure of hydrophilic amino acids (Sukop et al., 2025).

In addition, wet milling can be applied as a more cost- and time-efficient process, when utilizing maize grits (obtained from dry-fractionation) instead of the whole kernels. There was no significant reduction in starch recovery when steeping time was reduced to 6 h and SO_2_ concentration to 0.1% (88.9% dry base (d.b.), at a protein content of 0.54% d.b.). However, the maximum viscosity of starch is reduced by acid thinning as a result of the increased exposure of the grits to sulfure dioxide under acidic conditions (Eckhoff et al., 1993).

Besides maize starch, wet milling provides fiber fractions that primarily consist of different compositions of residual starch, hemicellulose, and cellulose (alongside with protein, oil, and lignin). Depending on the particle size, these serve as promising substrates for further modification by alkaline treatment or precipitation with ethanol, as well as enzymatic degradation of residual material and subsequent yeast fermentation. Such treatments can lead to the production of corn fiber gum and conversion into bioethanol. Alkaline treatments (1% and 2% (w/w) solutions) showed potential for obtaining high yields of hemicellulose from destarched maize fiber, thus improving enzymatic digestibility. Furthermore, residual material of high cellulose content has been suggested as a suitable substrate for baker’s yeast in the production of ethanol with a 90% yield (Gáspár et al., 2007). In order to reduce fibrous and starch-containing by-products of maize wet processing, it has been suggested to isolate corn fiber gum and starch as cross-linked conjugates for further applications. These conjugates possessed a low solubility (in water) compared to the highly soluble hemicellulose and proved to have a swelling index of 99.6%, due to cross-linking between AXs and starch. At concentrations of 14%, these conjugates could serve as binding agents in excipients and could prolong drug release for up to 12 h when used at high concentrations (57%) in tablets (Deokar et al., 2023).

This targeted utilization and valorization of wet-fractionated by-products beyond conventional starch production and their use as animal feed were further emphasized by Deepak and Jayadeep (2022), Paraskevopoulou et al. (2020), and Akin et al. (2025), who highlighted the great potential of maize germ, fiber, and gluten to serve as sources of functional, health-beneficial food ingredients, nutraceuticals, and for innovative biotechnological processes due to their rich content of bioactive compounds (tocols, phytosterols, carotenoids, phospholipids, AXs, xylooligosaccharides, polyphenols, vitamins). In this context, the bioavailability of these valuable compounds such as minerals and phenolic acids from underutilized maize processing products as well as the marked reduction of harmful mycotoxins could be promoted by *Bacillus subtilis* fermentation (Sun et al., 2025).

Moreover, recent studies by Peng et al. (2025) underlined the potential of zein extraction (depending on the applied method and solvent) from wet-milled starch-rich fraction to simultaneously tailor the functional properties (e.g., of swelling capacity, thermal viscosity, and water absorption) of the resulting starch residues.

Overall, the initial steeping process is undoubtedly a crucial step in the wet-fractionation, as it facilitates an efficient separation of maize biopolymers, particularly starch and proteins, while affecting their distribution, functionality, and structural properties as well as product yield and purity. These characteristics are specifically influenced by steeping time, type and concentration of acids, pH-value, presence and concentration of sulfur dioxide, temperature, and the addition of enzymes and lactic acid bacteria.

Wet-fractionation does not only provide valuable, high-purity products such as maize starch, suitable for direct application in foods, but also yields essential starting material for further extraction, industrial refining, and conversion into non-food products (e.g., bioethanol, pharmaceuticals, and fermentation substrate). However, despite the industrial relevance of this process, which primarily focuses on starch yield, only a limited number of studies have investigated the relationship between wet-fractionation and the functional properties of the resulting maize fractions, particularly fiber- and protein-rich by-products (Sukop et al., 2025). This highlights a significant gap in the current literature and provides an opportunity for further research in this area.

#### Wet-Fractionation Strategies in Rice Processing

Compared to maize, wet-fractionation of rice, for example, for starch production, is less commonly applied, which may be attributed to the more complex structural organization of the rice endosperm. However, due to its unique starch properties, various processes and strategies have been applied.

One of these approaches is alkaline soaking, which involves softening, cleaning, washing, milling with water, filtering, drying, sieving, or the application of wet grinding mills (e.g., continuously colloid or stone mill) (Serna-Saldivar, 2012). As previously mentioned, wet-fractionation causes significant amounts of waste water; nevertheless, it yields the lowest damaged starch content in rice flours, maintaining particle integrity and a narrow particle size distribution, compared to dry and semi-dry grinding methods (Yu et al., 2024). Soaking, also described as steeping, before wet-milling not only decreased damaged starch content, but also led to a leaching of proteins, lipids, and ash. Furthermore, extended soaking (7 days at 5 °C) compared to shorter rice incubation periods (7 h at 25 °C) resulted in reduced flour particle size and subsequently affected pasting properties (increased setback, breakdown, and final viscosities; declined pasting temperature) (Chiang & Yeh, 2002).

In addition to kernel steeping and conventional wet-milling, combined dry and wet-fractionations are increasingly applied in order to modify physico-chemical and nutritional properties of rice. Among these, the hydrothermal process of parboiling consists of the three consecutive steps: conditioning, heating, and drying of paddy rice. Initially, the moisture content of paddy rice (including the inedible husk) is increased to 30–35%, followed by a heating step using hot air, boiling, or pressure cooking with direct steam. Finally, the moisture of the paddy is reduced to 13–14% by drying. During this procedure, the high moisture of the paddy promotes starch gelatinization allowing the starch to act like a glue subsequently sealing microfissures or stress cracks. This results in increased head rice yield and kernel hardness thereby reducing the amount of broken kernels, enhanced storage stability, modified structural kernel appearance (glassy, slight discoloration, and reduced size), enhanced nutritional properties caused by the migration of, e.g., lipids, minerals, vitamin B from the bran (husk) to the endosperm, and improved functional characteristics including digestibility and water absorption. When not subjected to further milling after polishing and instead is cooked, parboiled rice exhibits improved shape retention, enhanced firmness, and greater fluffiness. It also demonstrates lower stickiness and a reduced loss of solids into the cooking water. These modifications in physicochemical properties are primarily attributed to the (partly irreversible) pre-gelatinization of starch, which leads to a reduction in molecular weight and crystallinity, as well as protein hydrolysis (increase in disulfide bonds), followed by enhanced viscosity and kernel hardness. However, parboiling represents an energy-intensive process, significantly affected by the amount of rice, the employed method, the variety and state of the rice kernels, and processing conditions such as soaking and steaming time (Muchlisyiyah et al., 2023).

Another wet-fractionation and extraction technique represents the US patent (No. US-3261690-A) protected solvent extraction milling (SEM), enabling the production of rice bran, oil, and debranned rice kernels (Rosentrater & Evers, 2018). Within this process, the brown rice is initially preconditioned or soaked with rice oil to soften the bran, followed by a wet-milling step using a rice oil-hexane mixture. The liquid phase facilitates the gentle removal and transport of the bran from the head rice, promotes the extraction of bran lipids into the solvent mixture, and contributes to reduced kernel breakage (increase of head rice yield up to 10%) at low processing temperatures compared to conventional dry milling methods. At the end of the process, debranned rice is separated and rinsed, bran and oil are separated by sedimentation, and hexane is finally removed from all products and recovered.

Moreover, SEM using non-polar solvents, such as hexane (characterized by high solubilizing capability, low boiling point and costs), enables the efficient recovery of high-purity rice bran oil from brown rice. The reduced content of residual fat in both bran and debranned rice kernels increases storage stability by reducing potential lipid oxidation. However, hexane, the predominant solvent in this conventional extraction process, is derived from non-renewable sources and poses environmental risks. Consequently, recent studies have focused on (partly) replacing hexane with bio-based alternatives such as ethanol, ethyl acetate, and isopropanol on applying innovative green technologies, including microwave, ultrasound-assisted extraction, supercritical carbon dioxide systems, or enzymes (Fraterrigo Garofalo et al., 2021).

Another rice fractionation method involving water application represents the semi-wet grinding, where brown rice is initially soaked, followed by drying of surface moisture and direct milling. This initial steeping step facilitates kernel softening, thereby reducing both milling time and energy input. Consequently, thermal and mechanical stresses during grinding are minimized, preserving the integrity of native polymer structures. Important parameters of the semi-wet milling process are soaking duration and temperature. Compared to the heat-intensive dry-milling, semi-wet grinding yielded significantly reduced starch damage (approximately 3%), although remained slightly higher (> 0.5%) than those observed for wet-grinding. Enhanced amounts of damaged starch notably declined pasting temperature and peak viscosity. Further, average particle size of starch granules was the highest using semi-wet milling (54 µm) in contrast to dry (35 µm) and wet-milling (8.5 µm), strongly influencing pasting (trough, peak viscosity) and thermal properties such as enthalpy of gelatinization, onset, peak, and completion temperature (Asmeda et al., 2016). Recent studies confirmed the reduction of starch damage by 32% applying wet-fractionation compared to dry-fractionation; however, the latter proved more effective (15%) in retaining health beneficial (antihypertensive and anxiolytic) compounds such as gamma-aminobutyric acid (GABA) in rice bran (Zhang et al., 2025).

In summary, although wet-fractionation of rice has played a minor role compared to maize or rice dry-fractionation, promising strategies, including soaking, parboiling, semi-dry milling, and SEM, have been applied and further optimized. These approaches enable reduced starch damage, enhanced nutritional, structural (kernel hardness and appearance), and functional (water absorption and digestibility) properties, and modified pasting behavior as well as a more efficient separation of the main constituents, respectively.

## Challenges and Future Work

The fractionation of maize and rice involves a highly complex process influenced and subsequently limited by a broad range of factors, including the type of cereal, its physicochemical properties, the chosen processing method and applied conditions, and the targeted yield and quality of the resulting fractions. A fundamental challenge in the design of dry-fractionation equipment, the selection of processing parameters, and the estimation of energy requirements lies in the specific cereal type and variety, as well as key physical (kernel size, weight, porosity, moisture) and mechanical (strength, stiffness, elasticity, plasticity) kernel properties, which are closely correlated with their chemical composition (Lyu et al., 2022; Purewal et al., 2022; Zhao et al., 2023). Consequently, these kernel variabilities limit the adaptability of existing milling equipment, as well as the (cost-intensive) operational optimization and standardization across multiple scales. Moreover, precise adjustment of fractionation parameters (including milling type, duration, and speed) represents a key technological challenge, as it aims to achieve an economically viable, high-yield, and accurate process, while enabling the production of stable, reproducible fractions with desired nutritional and functional properties, relevant for food and non-food applications (Blandino et al., 2013; Dominguez, 2021; Hafeez et al., 2015; Lyu et al., 2022; Qiu et al., 2019).

Wet-fractionation of maize represents a well-established process at lab-, pilot-, and industrial scale, with earlier research primarily focusing on steeping conditions (acid, SO_2_ source and concentration, temperature, duration) to improve protein solubilization and maximize subsequent starch recovery (Biss & Cogan, 1996; Singh et al., 1997; Yang et al., 2005). However, recent studies have focused on addressing the limited separation efficiency of starch from the outer cell wall layers, enhancing the functional properties of the biopolymers, and improving the accessibility of bioactive compounds through innovative enzymatic and physical treatments (Ozturk et al., 2021; Somavat et al., 2017; Sukop et al., 2025).

In contrast to maize, energy- and resource-intensive wet-fractionation is (still) less commonly applied due to the structurally more complex rice kernels, with parboiling representing the predominant strategy. Latest studies have demonstrated its potential to significantly improve starch pasting properties, nutrient migration into the endosperm, head rice yield, and overall cooking stability (Muchlisyiyah et al., 2023; Serna-Saldivar, 2012). Further, the simultaneous and relatively more efficient recovery of rice bran, debranned kernel, and especially oil applying SEM involves several key challenges such as solvent recovery and recycling to minimize environmental impact and process sustainability, as well as maintaining the overall quality of the extracted oil (Go et al., 2020; Kaimal et al., 2002).

Despite the industrial relevance of maize and rice fractionation processes, most studies have predominantly focused on the optimization of starch recovery and functionality, while the remaining fractions, often considered by-products, have received comparatively little attention. Addressing this notable gap in the current literature is crucial for optimizing processing conditions and fully exploiting the functional and economic potential of these underutilized fractions.

## Conclusion

**Fig. 1:**
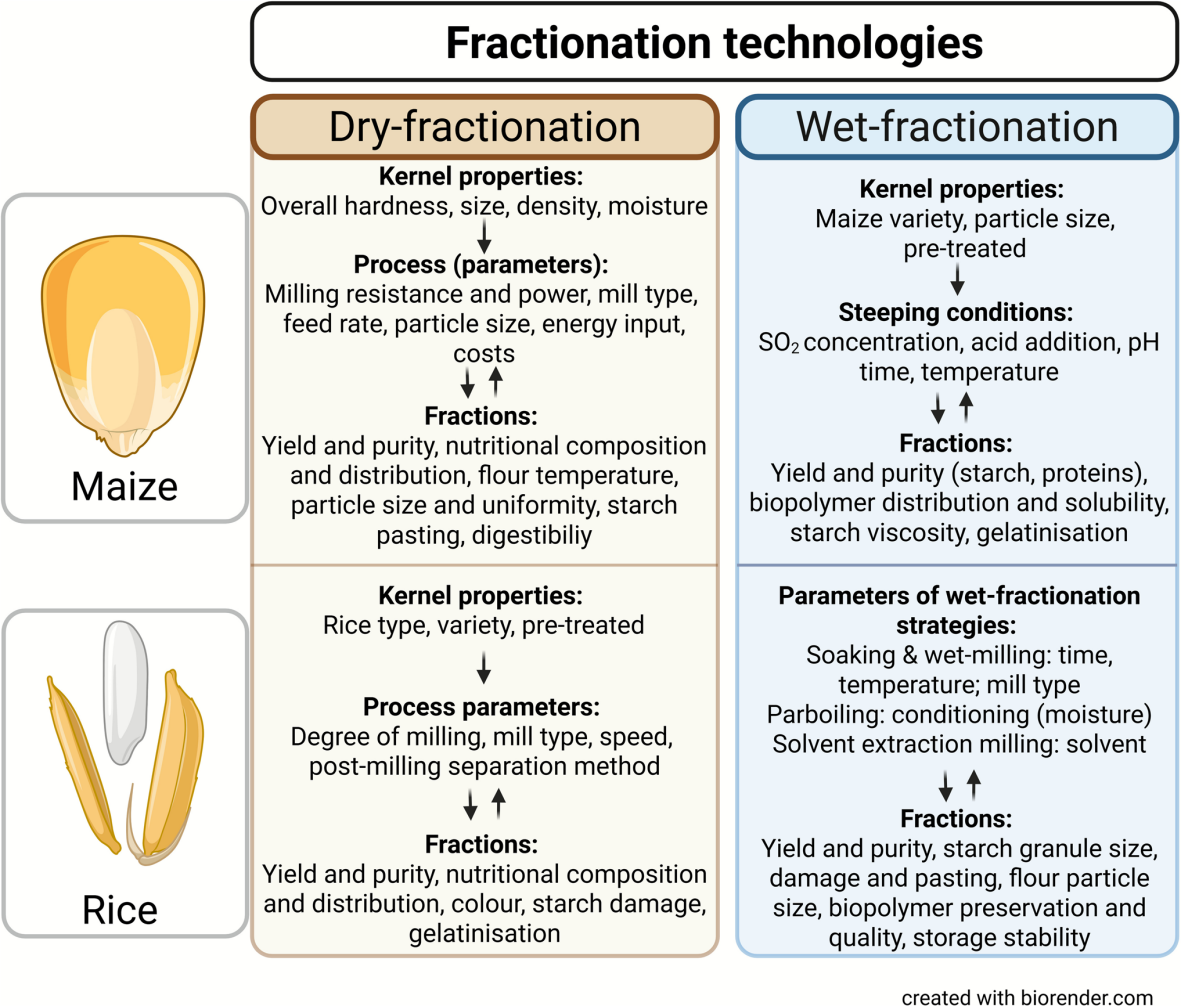
Schematic overview of maize and rice dry- and wet-fractionation strategies displaying the effects and relationships between kernel properties, process parameters, and resulting fraction characteristics

This review highlighted the potential of maize and rice fractions and biopolymers for further food and non-food applications, while emphasizing the crucial role of fractionation technologies and processing conditions on their purity, yield, and nutritional and functional quality (see Fig. 1). Moreover, a comparative analysis of these globally leading cereals revealed their distinct structural and compositional differences that determine the suitability and efficiency of fractionation techniques.

In maize dry-fractionation, intrinsic kernel properties (e.g., moisture) and maize types were identified as key determinants of process efficiency, with milling parameters, including milling type and speed, playing a crucial role in optimizing fraction yield and product characteristics. In contrast, efficient bran removal from head rice, strongly influenced by the time-dependent DOM, and subsequent milling steps as well as additional separation methods were found to be essential for modifying nutritional composition and thermal and pasting properties of fractions. Regarding maize wet-fractionation, the applied steeping conditions have been shown to be crucial not only for starch recovery and functionality, such as pasting characteristics, but also for the production of valuable by-products like corn fiber gum, which hold potential for application in innovative food products.

In rice processing, the combination of dry- and wet-fractionation offers significant opportunities to reduce starch damage and to modify starch granule size, influencing gelatinization properties. Moreover, parboiling and SEM were highlighted as innovative strategies to improve head rice kernel properties and polishing efficiency as well as enhanced bran removal and storage stability of fractions, respectively. However, a major limitation of these applications is the relatively high consumption of water.

Despite the wide range of available fractionation methods and applied conditions, further optimization steps remain essential to fully exploit the functional and nutritional potential of maize and rice. Additional research is needed to understand the complex interdependencies between kernel properties, process parameters, and the resulting biopolymer properties. A more comprehensive understanding of these interactions will support the development of modified and sustainable fractionation strategies as well as the production of tailored, innovative, and high-value fractions for targeted applications in the food and non-food sectors.

## Supplementary Information

Below is the link to the electronic supplementary material.

ESM 1 (DOCX 19.0 KB)

## Data Availability

No datasets were generated or analysed during the current study.

## References

[CR1] AACC International. (2010). *Approved Methods of Analysis* (11th ed.). AACC International: St. Paul, MN, USA.

[CR2] Abdel-Aal, E.-S. M. (2024). Insights into grain milling and fractionation practices for improved food sustainability with emphasis on wheat and peas. *Foods*. 10.3390/foods13101532 10.3390/foods13101532 PMC11121700 38790832

[CR3] Abiose Sumbo, H., & Victor, I. A. (2014). Comparison of chemical composition, functional properties and amino acids composition of quality protein maize and common maize (*Zea may* L). *African Journal of Food Science and Technology,**5*(3), 81–89.

[CR4] Akin, M., Jukic, M., Lukinac, J., Yilmaz, B., Özogul, F., & Rocha, J. M. (2025). *Valorization and functionalization of cereal-based industry by-products for nutraceuticals* (pp. 173–222). Nutraceutics from Agri-Food By-Products. 10.1002/9781394174867.ch6

[CR5] Al-Doury, M. K. W., Hettiarachchy, N. S., & Horax, R. (2018). Rice-endosperm and rice-bran proteins: A review. *Journal of the American Oil Chemists’ Society,**95*(8), 943–956. 10.1002/aocs.12110

[CR6] Ali, A. A. (2021). Maize productivity in the new millennium. In H. Awaad, M. Abu-hashim, & A. Negm (Eds.), *Mitigating environmental stresses for agricultural sustainability in Egypt* (pp. 509–535). Springer International Publishing. 10.1007/978-3-030-64323-2_19

[CR7] Amagliani, L., O’Regan, J., Kelly, A. L., & O’Mahony, J. A. (2017). The composition, extraction, functionality and applications of rice proteins: A review. *Trends in Food Science & Technology,**64*, 1–12. 10.1016/j.tifs.2017.01.008

[CR8] Arzami, A. N., Ho, T. M., & Mikkonen, K. S. (2022). Valorization of cereal by-product hemicelluloses: Fractionation and purity considerations. *Food Research International,**151*, 110818. 10.1016/j.foodres.2021.110818 34980370 10.1016/j.foodres.2021.110818

[CR9] Asmeda, R., Noorlaila, A., & Norziah, M. H. (2016). Relationships of damaged starch granules and particle size distribution with pasting and thermal profiles of milled MR263 rice flour. *Food Chemistry,**191*, 45–51. 10.1016/j.foodchem.2015.05.095 26258700 10.1016/j.foodchem.2015.05.095

[CR10] Bala, B. K. (2020). *Agro-product processing technology*. CRC Press. 10.1201/9780429487507

[CR11] Barrera-Arellano, D., Badan-Ribeiro, A. P., & Serna-Saldivar, S. O. (2019). Chapter 21 - Corn oil: Composition, processing, and utilization. In S. O. Serna-Saldivar (Ed.), *Corn* (3rd ed., pp. 593–613). AACC International Press. 10.1016/B978-0-12-811971-6.00021-8

[CR12] Bastos, R., Coelho, E., & Coimbra, M. A. (2018). 8 - Arabinoxylans from cereal by-products: Insights into structural features, recovery, and applications. In C. M. Galanakis (Ed.), *Sustainable recovery and reutilization of cereal processing by-products: Woodhead publishing series in food science, technology and nutrition* (pp. 227–251). Woodhead Publishing. 10.1016/B978-0-08-102162-0.00008-3

[CR13] Bean, S. R., Akin, P. A., & Aramouni, F. M. (2021). Zein functionality in viscoelastic dough for baked food products. *Journal of Cereal Science,**100*, 103270. 10.1016/j.jcs.2021.103270

[CR14] Biss, R., & Cogan, U. (1996). Sulfur dioxide in acid environment facilitates corn steeping. *Cereal Chemistry, 73*(1), S. 40–44.

[CR15] Blandino, M., Alfieri, M., Giordano, D., Vanara, F., & Redaelli, R. (2017). Distribution of bioactive compounds in maize fractions obtained in two different types of large scale milling processes. *Journal of Cereal Science,**77*, 251–258. 10.1016/j.jcs.2017.08.006

[CR16] Blandino, M., Sacco, D., & Reyneri, A. (2013). Prediction of the dry-milling performance of maize hybrids through hardness-associated properties. *Journal of the Science of Food and Agriculture,**93*(6), 1356–1364. 10.1002/jsfa.5897 23139165 10.1002/jsfa.5897

[CR17] Bresciani, A., Giordano, D., Vanara, F., Blandino, M., & Marti, A. (2021). The effect of the amylose content and milling fractions on the physico-chemical features of co-extruded snacks from corn. *Food Chemistry,**343*, Article 128503. 10.1016/j.foodchem.2020.128503 33243562 10.1016/j.foodchem.2020.128503

[CR18] Brites, P., Luís, J., Kapusniak, K., Wojcik, M., Nunes, C., Coimbra, M. A., et al. (2025). Starch-rich rice by-products as a renewable resource for sustainable production of flexible, water tolerant, antioxidant, and UV-protective bioplastics. *Advanced Sustainable Systems, e00278*. 10.1002/adsu.202500278

[CR19] Budăcan, I., Drocaș, I., & Pop, D. (2013). Influence of grains moisture content on milling parameters. *Acta Technica Napocensis - Series: Applied Mathematics, Mechanics, and Engineering; Vol 56, No 2 (2013): Acta Technica Napocensis - Series: Applied Mathematics and Mechanics*. https://atna-mam.utcluj.ro/index.php/Acta/article/view/87. Accessed 17 Dec 2024

[CR20] Cai, J., Man, J., Huang, J., Liu, Q., Wei, W., & Wei, C. (2015). Relationship between structure and functional properties of normal rice starches with different amylose contents. *Carbohydrate Polymers*. 10.1016/j.carbpol.2015.02.067 25857957 10.1016/j.carbpol.2015.02.067

[CR21] Cai, Z., Wei, Y., Zhang, H., Rao, P., & Wang, Q. (2021). Holistic review of corn fiber gum: Structure, properties, and potential applications. *Trends in Food Science & Technology,**111*, 756–770. 10.1016/j.tifs.2021.03.034

[CR22] Cao, X., Wen, H., Li, C., & Gu, Z. (2009). Differences in functional properties and biochemical characteristics of congenetic rice proteins. *Journal of Cereal Science,**50*(2), 184–189. 10.1016/j.jcs.2009.04.009

[CR23] Chandi, G. K., & Sogi, D. S. (2007). Functional properties of rice bran protein concentrates. *Journal of Food Engineering,**79*(2), 592–597. 10.1016/j.jfoodeng.2006.02.018

[CR24] Chen, Z., Li, P., Ji, Q., Xing, Y., Ma, X., & Xia, Y. (2023). All-polysaccharide composite films based on calcium alginate reinforced synergistically by multidimensional cellulose and hemicellulose fractionated from corn husks. *Materials Today Communications,**34*, Article 105090. 10.1016/j.mtcomm.2022.105090

[CR25] Chiang, P.-Y., & Yeh, A.-I. (2002). Effect of soaking on wet-milling of rice. *Journal of Cereal Science,**35*(1), 85–94. 10.1006/jcrs.2001.0419

[CR26] Cornejo-Ramírez, Y. I., Martínez-Cruz, O., Del Toro-Sánchez, C. L., Wong-Corral, F. J., Borboa-Flores, J., & Cinco-Moroyoqui, F. J. (2018). The structural characteristics of starches and their functional properties. *CyTA - Journal of Food,**16*(1), 1003–1017. 10.1080/19476337.2018.1518343

[CR27] Dang, T. T., & Vasanthan, T. (2019). Modification of rice bran dietary fiber concentrates using enzyme and extrusion cooking. *Food Hydrocolloids,**89*, 773–782. 10.1016/j.foodhyd.2018.11.024

[CR28] David, O. A., Daniel, E. E., Agbenu, A. C., Nyerere, A. C., Praise, A. J., & Ape, S. (2022). Nutritional composition and functional properties of maize – Soya bean composite flour. *Global Journal of Research in Chemistry and Pharmacy,**1*(1), 7–17. 10.58175/gjrcp.2022.1.1.0022

[CR29] de La Hera, E., Talegón, M., Caballero, P., & Gómez, M. (2013). Influence of maize flour particle size on gluten-free breadmaking. *Journal of the Science of Food and Agriculture,**93*(4), 924–932. 10.1002/jsfa.5826 22886488 10.1002/jsfa.5826

[CR30] Deepak, T. S., & Jayadeep, P. A. (2022). Prospects of maize (corn) wet milling by-products as a source of functional food ingredients and nutraceuticals. *Food Technology and Biotechnology,**60*(1), 109–120. 10.17113/ftb.60.01.22.7340 35440878 10.17113/ftb.60.01.22.7340 PMC8990988

[CR31] Delcour, J. A., & Hoseney, R. C. (2010). *Principles of Cereal Science and Technology* (3rd ed.). St. Paul, MN, USA: AACC International.

[CR32] Deokar, G. S., Deokar, A. M., Kshirsagar, S. J., Buranasompob, A., & Nirmal, N. P. (2023). Extraction, physicochemical characterization, functionality, and excipient ability of corn fiber gum-starch isolate from corn milling industry waste. *International Journal of Pharmaceutics,**645*, Article 123401. 10.1016/j.ijpharm.2023.123401 37696343 10.1016/j.ijpharm.2023.123401

[CR33] Devi, V., Sethi, M., Kaur, C., Singh, V., Kumar, R., & Chaudhary, D. P. (2024). Temporal profile of amino acids and protein fractions in the developing kernel of maize germplasm. *Scientific Reports*. 10.1038/s41598-024-65514-2 39511239 10.1038/s41598-024-65514-2 PMC11543656

[CR34] Dominguez, E. N. (2021). Design, fabrication, and performance evaluation of a hammer mill for small-scale corn milling operation. *International Research Journal of Innovations in Engineering and Technology,**5*(9), 46.

[CR35] Donmez, D., Pinho, L., Patel, B., Desam, P., & Campanella, O. H. (2021). Characterization of starch–water interactions and their effects on two key functional properties: Starch gelatinization and retrogradation. *Current Opinion in Food Science,**39*, 103–109. 10.1016/j.cofs.2020.12.018

[CR36] Eckhoff, S. R., Jayasena, W. V., & Spillman, C. K. (1993). Wet milling of maize grits. *Cereal Chemistry,**70*, 257.

[CR37] Erenstein, O., Jaleta, M., Sonder, K., Mottaleb, K., & Prasanna, B. M. (2022). Global maize production, consumption and trade: Trends and R&D implications. *Food Security,**14*(5), 1295–1319. 10.1007/s12571-022-01288-7

[CR38] Esa, N. M., Ling, T. B., & Peng, L. S. (2013). By-products of rice processing: An overview of health benefits and applications. *Journal of Rice Research, **1*(107). 10.4172/jrr.1000107

[CR39] Farooq, M. A., & Yu, J. (2025). Starches in rice: Effects of rice variety and processing/cooking methods on their glycemic index.* Foods*. 10.3390/foods14122022 10.3390/foods14122022 PMC12192320 40565633

[CR40] Fraterrigo Garofalo, S., Tommasi, T., & Fino, D. (2021). A short review of green extraction technologies for rice bran oil. *Biomass Conversion and Biorefinery,**11*(2), 569–587. 10.1007/s13399-020-00846-3

[CR41] Gáspár, M., Kálmán, G., & Réczey, K. (2007). Corn fiber as a raw material for hemicellulose and ethanol production. *Process Biochemistry,**42*(7), 1135–1139. 10.1016/j.procbio.2007.04.003

[CR42] Go, A. W., Pham, T. Y. N., Truong, C. T., Quijote, K. L., Angkawijaya, A. E., Agapay, R. C., et al. (2020). Improved solvent economy and rate of rice bran lipid extraction using hydrolyzed rice bran with hexane as solvent. *Biomass and Bioenergy,**142*, Article 105773. 10.1016/j.biombioe.2020.105773

[CR43] Godoy, MRCde., Kerr, K. R., & Fahey, G. C., Jr. (2013). Alternative dietary fiber sources in companion animal nutrition. *Nutrients,**5*(8), 3099–3117. 10.3390/nu5083099 23925042 10.3390/nu5083099 PMC3775244

[CR44] Grossmann, L., & McClements, D. J. (2023). Current insights into protein solubility: A review of its importance for alternative proteins. *Food Hydrocolloids,**137*, Article 108416. 10.1016/j.foodhyd.2022.108416

[CR45] Hafeez, A., Mader, A., Röhe, I., Ruhnke, I., Boroojeni, F. G., Yousaf, M. S., Männer, K., & Zentek, J. (2015). Effect of milling method, thermal treatment, and particle size of feed on exterior and interior egg quality in laying hens. *European Journal of Poultry Science*, *79*. 10.3382/ps/peu070 25630675

[CR46] Hu, W.-X., Chen, J., Xu, F., Chen, L., & Zhao, J.-W. (2020). Study on crystalline, gelatinization and rheological properties of japonica rice flour as affected by starch fine structure. *International Journal of Biological Macromolecules,**148*, 1232–1241. 10.1016/j.ijbiomac.2019.11.020 31759021 10.1016/j.ijbiomac.2019.11.020

[CR47] Hussain, M., Qamar, A., Saeed, F., Rasheed, R., Niaz, B., Afzaal, M., Mushtaq, Z., & Anjum, F. (2021). Biochemical properties of maize bran with special reference to different phenolic acids. *International Journal of Food Properties,**24*(1), 1468–1478. 10.1080/10942912.2021.1973026

[CR48] Jaichakan, P., Nakphaichit, M., Rungchang, S., Weerawatanakorn, M., Phongthai, S., & Klangpetch, W. (2021). Two-stage processing for xylooligosaccharide recovery from rice by-products and evaluation of products: Promotion of lactic acid-producing bacterial growth and food application in a high-pressure process. *Food Research International,**147*, Article 110529. 10.1016/j.foodres.2021.110529 34399507 10.1016/j.foodres.2021.110529

[CR49] Jayadeep, A., Singh, V., Sathyendra Rao, B. V., Srinivas, A., & Ali, S. Z. (2009). Effect of physical processing of commercial de-oiled rice bran on particle size distribution, and content of chemical and bio-functional components. *Food and Bioprocess Technology,**2*(1), 57–67. 10.1007/s11947-008-0094-6

[CR50] Jayaprakash, G., Bains, A., Chawla, P., Fogarasi, M., & Fogarasi, S. (2022). A narrative review on rice proteins: Current scenario and food industrial application. *Polymers*. 10.3390/polym14153003 35893967 10.3390/polym14153003 PMC9370113

[CR51] Jiang, F., Shen, W., Peng, D., Jin, W., & Huang, Q. (2024). Self-assembly of rice proteins: A perspective on elevating rice protein techno-functional properties. *Trends in Food Science & Technology*. 10.1016/j.tifs.2024.104624

[CR52] Kaimal, T. N. B., Vali, S. R., Rao, B. V. S. K., Chakrabarti, P. P., Vijayalakshmi, P., Kale, V., et al. (2002). Origin of problems encountered in rice bran oil processing. *European Journal of Lipid Science and Technology,**104*(4), S. 203–211. 10.1002/1438-9312(200204)104:4<203::AID-EJLT203>3.0.CO;2-X. Accessed 5 May 2025

[CR53] Kalpanadevi, C., Singh, V., & Subramanian, R. (2018). Influence of milling on the nutritional composition of bran from different rice varieties. *Journal of Food Science and Technology,**55*(6), 2259–2269. 10.1007/s13197-018-3143-9 29892126 10.1007/s13197-018-3143-9 PMC5976611

[CR54] Kaur, A., Virdi, A. S., Singh, N., Singh, A., & Kaler, R. S. S. (2021). Effect of degree of milling and defatting on proximate composition, functional and texture characteristics of gluten-free muffin of bran of long-grain *indica* rice cultivars. *Food Chemistry,**345*, Article 128861. 10.1016/j.foodchem.2020.128861 33348134 10.1016/j.foodchem.2020.128861

[CR55] Keya, A. C., Al Mamun, M. R., Hossen, M. A., Alim, M. S., Hossain, B., Baidya, J. (2025): Drying and tempering effect on milling process of paddy in unparboiled condition: Drying and tempering effect on milling process of paddy. *Agricultural Engineering International: CIGR Journal* 27 (1).

[CR56] Khairuddin, M. A., & Lasekan, O. (2021). Gluten-free cereal products and beverages: A review of their health benefits in the last five years. *Foods*. 10.3390/foods10112523 10.3390/foods10112523 PMC8618534 34828804

[CR57] Kumar, A., Joshi, R., Awasthi, T., & Singh, N. (2024). Insights into the non-targeted metabolomic profile, rheological, functional and structural characteristics of rice bran and pulse protein isolates. *International Journal of Food Science and Technology,**59*(1), 560–572. 10.1111/ijfs.16497

[CR58] Kumar, M., Tomar, M., Potkule, J., Verma, R., Punia, S., Mahapatra, A., Belwal, T., Dahuja, A., Joshi, S., Berwal, M. K., Satankar, V., Bhoite, A. G., Amarowicz, R., Kaur, C., & Kennedy, J. F. (2021). Advances in the plant protein extraction: Mechanism and recommendations. *Food Hydrocolloids, 115*, 106595. 10.1016/j.foodhyd.2021.106595

[CR59] Kurek, M., & Wyrwisz, J. (2015). The application of dietary fiber in bread products. *Journal of Food Processing and Technology,**6*(5), 447–450.

[CR60] Landry, J., Delhaye, S., & Di Gioia, L. (1999). Protein distribution in gluten products isolated during and after wet-milling of maize grains. *Cereal Chemistry,**76*(4), 503–505. 10.1094/CCHEM.1999.76.4.503

[CR61] Li, J., Kong, X., & Ai, Y. (2022). Modification of granular waxy, normal and high-amylose maize starches by maltogenic α-amylase to improve functionality. *Carbohydrate Polymers*. 10.1016/j.carbpol.2022.119503 35550756 10.1016/j.carbpol.2022.119503

[CR62] Li, W., Yu, Y., Wang, L., Luo, Y., Peng, Y., Xu, Y., et al. (2021). The genetic architecture of the dynamic changes in grain moisture in maize. *Plant Biotechnology Journal*. 10.1111/pbi.13541 33386670 10.1111/pbi.13541 PMC8196655

[CR63] Liang, D., Luo, W., Xu, M., Guo, D., Guo, J., Hu, Y., et al. (2025). The effect of high-amylose maize starch on the digestibility of wheat starch after high-temperature cooking. *International Journal of Biological Macromolecules*. 10.1016/j.ijbiomac.2025.143258 40250662 10.1016/j.ijbiomac.2025.143258

[CR64] Lisboa, H. M., Andrade, R., & Sarinho, A. M. M. (2025). Milling and dry fractionation. *In Cereal Grains* (1st ed., pp. 1–20). CRC Press.

[CR65] Liu, J., Wu, Y., Chen, H., An, H., Liu, Y., & Xu, J. (2022). Effect of the degree of milling on the microstructure and composition of japonica rice. *Grain & Oil Science and Technology,**5*(4), 194–203. 10.1016/j.gaost.2022.09.002

[CR66] Liu, J., Yu, X.-S., Wang, Y.-D., Fang, G.-H., & Liu, Y.-W. (2020). A cleaner approach for corn starch production by ultrasound-assisted laboratory scale wet-milling. *Food Science and Technology Research,**26*, 469–478. 10.3136/fstr.26.469

[CR67] Liu, K., Zheng, J., & Chen, F. (2017). Relationships between degree of milling and loss of Vitamin B, minerals, and change in amino acid composition of brown rice. *LWT,**82*, 429–436. 10.1016/j.lwt.2017.04.067

[CR68] Liu, Y., Chen, X., Xu, Y., Xu, Z., Li, H., Sui, Z., et al. (2021). Gel texture and rheological properties of normal amylose and waxy potato starch blends with rice starches differing in amylose content. *International Journal of Food Science and Technology*. 10.1111/ijfs.14826

[CR69] Liu, Y., Zhang, H., Yi, C., Quan, K., & Lin, B. (2021). Chemical composition, structure, physicochemical and functional properties of rice bran dietary fiber modified by cellulase treatment. *Food Chemistry,**342*, Article 128352. 10.1016/j.foodchem.2020.128352 33268168 10.1016/j.foodchem.2020.128352

[CR70] Louis, A. C. F., Venkatachalam, S., & Gupta, S. (2022). Innovative strategy for rice straw valorization into nanocellulose and nanohemicellulose and its application. *Industrial Crops and Products,**179*, Article 114695. 10.1016/j.indcrop.2022.114695

[CR71] Luo, X., Cheng, B., Zhang, W., Shu, Z., Wang, P., & Zeng, X. (2021). Structural and functional characteristics of japonica rice starches with different amylose contents. *CyTA - Journal of Food*. 10.1080/19476337.2021.1927194

[CR72] Lv, X., Hong, Y., Zhou, Q., & Jiang, C. (2021). Structural features and digestibility of corn starch with different amylose content. *Frontiers in Nutrition,**8*, Article 692673. 10.3389/fnut.2021.692673 34235171 10.3389/fnut.2021.692673 PMC8257001

[CR73] Lyu, F., Hendriks, W. H., van der Poel, A., & Thomas, M. (2022). Particle size distribution, energy consumption, nutrient composition and in vitro ileal digestion characteristics of hammer milled maize and soybean meal affected by moisture content. *Animal Feed Science and Technology,**288*, 115317. 10.1016/j.anifeedsci.2022.115317

[CR74] Ma, Z., Mondor, M., Dowle, A. A., Goycoolea, F. M., & Hernández-Álvarez, A. J. (2025). Buffalo worm (*Alphitobius diaperinus*) proteins: Structural properties, proteomics and nutritional benefits. *Food Chemistry*. 10.1016/j.foodchem.2024.141757 39503093 10.1016/j.foodchem.2024.141757

[CR75] Mendonça, S., Grossmann, M., & Verhé, R. (2000). Corn bran as a fibre source in expanded snacks. *LWT,**33*(1), 2–8. 10.1006/fstl.1999.0601

[CR76] Mohidem, N. A., Hashim, N., Shamsudin, R., & Che Man, H. (2022). Rice for food security: Revisiting its production, diversity, rice milling process and nutrient content. *Agriculture*. 10.3390/agriculture12060741

[CR77] Moreno-Zaragoza, J., Pecina-Ornelas, D. F., Agama-Acevedo, E., Rosell, C. M., & Bello-Pérez, L. A. (2025). High amylose maize starch can produce an inclusion complex with extract of medicinal plants of *Amphipterygium adstringent*. *Cereal Chemistry*. 10.1002/cche.10866

[CR78] Mounir, S., Ghandour, A., Shatta, A., & Farid, E. (2024). Starch: Properties and functionality. In (pp. 1–46). 10.1201/9781032655598-1

[CR79] Muchlisyiyah, J., Shamsudin, R., Kadir Basha, R., Shukri, R., How, S., Niranjan, K., & Onwude, D. (2023). Parboiled rice processing method, rice quality, health benefits, environment, and future perspectives: A review. *Agriculture*. 10.3390/agriculture13071390

[CR80] Mule, T. A., Sawant, S. S., & Odaneth, A. A. (2024). Maize bran as a potential substrate for production of β-glucosidase. *Biomass Conversion and Biorefinery,**14*(3), 4029–4039. 10.1007/s13399-022-02747-z

[CR81] Mut, Z., Kardeş, Y. M., & Erbaş Köse, Ö. D. (2022). Determining the grain yield and nutritional composition of maize cultivars in different growing groups. *Turkish Journal of Field Crops,**27*(1), 158–166. 10.17557/tjfc.1107691

[CR82] Ngamnikom, P., & Songsermpong, S. (2011). The effects of freeze, dry, and wet grinding processes on rice flour properties and their energy consumption. *Journal of Food Engineering,**104*(4), 632–638. 10.1016/j.jfoodeng.2011.02.001

[CR83] Nikolić, V., Simić, M., Žilić, S., Vasić, M., Milovanović, D., & Sarić, B. (2023). Influence of particle size distribution on in vitro digestibility and nutritional quality of differently coloured wholegrain maize flours. *Journal of Food and Nutrition Research, **62*, 245–235.

[CR84] Osborne, T. B. (1924). *The vegetable proteins*. Longmans, Green and Company.

[CR85] Ozcalik, O., & Tihminlioglu, F. (2013). Barrier properties of corn zein nanocomposite coated polypropylene films for food packaging applications. *Journal of Food Engineering,**114*(4), 505–513. 10.1016/j.jfoodeng.2012.09.005

[CR86] Ozturk, O. K., Kaasgaard, S. G., Palmén, L. G., Vidal, B., & Hamaker, B. R. (2021). Protein matrix retains most starch granules within corn fiber from corn wet-milling process. *Industrial Crops and Products,**165*, Article 113429. 10.1016/j.indcrop.2021.113429

[CR87] Paiva, F. F., Vanier, N. L., Berrios, J. D. J., Pan, J., Villanova, Fd. A., Takeoka, G., & Elias, M. C. (2014). Physicochemical and nutritional properties of pigmented rice subjected to different degrees of milling. *Journal of Food Composition and Analysis,**35*(1), 10–17. 10.1016/j.jfca.2014.05.003

[CR88] Paraskevopoulou, A., Anagnostara, I., Bezati, G., Rizou, T., Pavlidou, E., Vouvoudi, E., & Kiosseoglou, V. (2020). Water extraction residue from maize milling by-product as a potential functional ingredient for the enrichment with fibre of cakes. *LWT,**129*, Article 109604. 10.1016/j.lwt.2020.109604

[CR89] Pareyt, B., Finnie, S. M., Putseys, J. A., & Delcour, J. A. (2011). Lipids in bread making: Sources, interactions, and impact on bread quality. *Journal of Cereal Science*. 10.1016/j.jcs.2011.08.011

[CR90] Paulik, S., Jekle, M., & Becker, T. (2019). Mechanically and thermally induced degradation and modification of cereal biopolymers during grinding. *Polymers*. 10.3390/polym11030448 10.3390/polym11030448 PMC6473319 30960432

[CR91] Peanparkdee, M., & Iwamoto, S. (2019). Bioactive compounds from by-products of rice cultivation and rice processing: Extraction and application in the food and pharmaceutical industries. *Trends in Food Science & Technology,**86*, 109–117. 10.1016/j.tifs.2019.02.041

[CR92] Peng, Y., Wu, Y., Shan, Z., Wen, X., Li, M., & Ni, Y. (2025). Preparation and characterization of corn starch fractions from varied zein extraction processes: Implications for industrial structure adjustment. *Food and Bioprocess Technology*. 10.1007/s11947-025-03901-x

[CR93] Pérez, O. E., Haros, M., & Suarez, C. (2001). Corn steeping: Influence of time and lactic acid on isolation and thermal properties of starch. *Journal of Food Engineering,**48*(3), 251–256. 10.1016/S0260-8774(00)00165-5

[CR94] Praseptiangga, D., Rahmawati, A., Manuhara, G. J., Khasanah, L. U., & Utami, R. (2021). Effects of plasticizer and cinnamon essential oil incorporation on mechanical and water barrier properties of semirefined iota-carrageenan-based edible film. *IOP Conference Series: Earth and Environmental Science,**828*(1), 12034. 10.1088/1755-1315/828/1/012034

[CR95] Pulivarthi, M., Buenavista, R. M., Punia Bangar, S., Li, Y., Pordesimo, L., Bean, S., & Siliveru, K. (2023). Dry-fractionation process operations in the production of protein concentrates: A review. *Comprehensive Reviews in Food Science and Food Safety, 22*. 10.1111/1541-4337.13237 10.1111/1541-4337.13237 37779384

[CR96] Punia, S., Kumar, M., Sandhu, K. S., & Whiteside, W. S. (2021). Rice-bran oil: An emerging source of functional oil. *Journal of Food Processing and Preservation,**45*(4), e15318. 10.1111/jfpp.15318

[CR97] Purewal, S. S., Kaur, P., Bangar, S. P., Sandhu, K. S., Singh, S. K., & Kaur, M. (2022). Maize: Nutritional composition, processing, and industrial uses. *Cereals*. CRC Press. https://books.google.at/books?id=NSyEEAAAQBAJ. Accessed 10 Jul 2024

[CR98] Qamar, S., Manrique, Y. J., Parekh, H., & Falconer, J. R. (2020). Nuts, cereals, seeds and legumes proteins derived emulsifiers as a source of plant protein beverages: A review. *Critical Reviews in Food Science and Nutrition*. 10.1080/10408398.2019.1657062 10.1080/10408398.2019.1657062 31478387

[CR99] Qiu, C., Li, P., Li, Z., Corke, H., & Sui, Z. (2019). Combined speed and duration of milling affect the physicochemical properties of rice flour. *Food Hydrocolloids,**89*, 188–195. 10.1016/j.foodhyd.2018.10.042

[CR100] Rashwan, A. K., Osman, A. I., Abdelshafy, A. M., Mo, J., & Chen, W. (2025). Plant-based proteins: Advanced extraction technologies, interactions, physicochemical and functional properties, food and related applications, and health benefits. *Critical Reviews in Food Science and Nutrition,**65*(4), 667–694. 10.1080/10408398.2023.2279696 37966163 10.1080/10408398.2023.2279696

[CR101] Rondanelli, M., Miccono, A., Peroni, G., Nichetti, M., Infantino, V., Spadaccini, D., Alalwan, T. A., Faliva, M. A., & Perna, S. (2021). Rice germ macro-and micronutrients: A new opportunity for the nutraceutics. *Natural Product Research,**35*(9), 1532–1536. 31478776 10.1080/14786419.2019.1660329

[CR102] Ronie, M. E., Abdul Aziz, A. H., Mohd Noor, N. Q., Yahya, F., & Mamat, H. (2022). Characterisation of bario rice flour varieties: Nutritional compositions and physicochemical properties. *Applied Sciences*. 10.3390/app12189064

[CR103] Ronie, M. E., & Mamat, A. H. (2022). Factors affecting the properties of rice flour: A review. *Food Research*. 10.26656/fr.2017.6(6).531

[CR104] Rose, D. J., Inglett, G. E., & Liu, S. X. (2010). Utilisation of corn (*Zea mays*) bran and corn fiber in the production of food components. *Journal of the Science of Food and Agriculture,**90*(6), 915–924. 10.1002/jsfa.3915 20355130 10.1002/jsfa.3915

[CR105] Rosentrater, K. A., & Evers, A. D. (2018). Chapter 14 - Wet milling: Separating starch, gluten (protein) and fibre. *Kent’s Technology of Cereals* (pp. 839–860). Elsevier. 10.1016/B978-0-08-100529-3.00014-1

[CR106] Roye, C., Bulckaen, K., de Bondt, Y., Liberloo, I., van de Walle, D., Dewettinck, K., & Courtin, C. M. (2020). Side-by-side comparison of composition and structural properties of wheat, rye, oat, and maize bran and their impact on in vitro fermentability. *Cereal Chemistry,**97*(1), 20–33. 10.1002/cche.10213

[CR107] Sayaslan, A., Koyuncu, M., & Şahin, N. (2023). Chapter 27 - Wet-milling of wheat and maize. In ICC (Ed.), *ICC Standards: 21*^*st*^* edition* (pp. 261–268). Elsevier. 10.1016/B978-0-323-95295-8.00025-3

[CR108] Schirmer, M., Jekle, M., & Becker, T. (2015). Starch gelatinization and its complexity for analysis. *Starch - Stärke,**67*(1–2), 30–41. 10.1002/star.201400071

[CR109] Serna-Saldivar, S. O. (2012). *Cereal grains*. CRC Press. https://ebookcentral.proquest.com/lib/ubhohenheim/detail.action?docID=1449130#goto_toc. 10.1201/b11726. Accessed 22 Oct 2024

[CR110] Serna-Saldivar, S. O. (Ed.). (2019). *Corn* (3rd ed.). AACC International Press.

[CR111] Shandera, D. L., & Jackson, D. S. (1996). Effect of corn wet-milling conditions (sulfur dioxide, lactic acid, and stepping temperature) on starch functionality. *Cereal Chemistry,**73*(5), 632–637.

[CR112] Sharif, M. K., Butt, M. S., Anjum, F. M., & Khan, S. H. (2014). Rice bran: A novel functional ingredient. *Critical Reviews in Food Science and Nutrition,**54*(6), 807–816. 10.1080/10408398.2011.608586 24345050 10.1080/10408398.2011.608586

[CR113] Shin, M., Baek, M., No, J., & Mun, S. (2022). Effect of different degrees of milling on the protein composition in brown rice brans. *Journal of Food Measurement and Characterization,**16*(1), 214–221. 10.1007/s11694-021-01144-w

[CR114] Sidhu, G. K., Singh, A. K., & Singh, M. (2016). Effect of milling speed on the quality and storage stability of maize flour. *Journal of Applied and Natural Science,**8*(2), 1015–1021.

[CR115] Silventoinen, P., Rommi, K., Holopainen-Mantila, U., Poutanen, K., & Nordlund, E. (2019). Biochemical and techno-functional properties of protein- and fibre-rich hybrid ingredients produced by dry-fractionation from rice bran. *Food and Bioprocess Technology,**12*(9), 1487–1499. 10.1007/s11947-019-02307-w

[CR116] Singh, H., Lin, J.-H., Huang, W.-H., & Chang, Y.-H. (2012). Influence of amylopectin structure on rheological and retrogradation properties of waxy rice starches. *Journal of Cereal Science*. 10.1016/j.jcs.2012.04.007

[CR117] Singh, S. K., Johnson, La., Pollak, L. M., Fox, S. R., & Bailey, T. B. (1997). Comparison of laboratory and pilot-plant corn wet-milling procedures. *Cereal Chemistry,**74*(1), 40–48.

[CR118] Singh, S. N., Srivastav, S., & Sahu, F. M. (2018). Fundamentals of food engineering and applications: Chapter 5: Milling technology of grains. In S. Srivastav, P. M. Ganorkar (Eds.), *Fundamentals of food engineering and applications.* Vardhan Gupta. https://www.researchgate.net/publication/348297955_Milling_Technology_of_Grains. Accessed 25 Jun 2024

[CR119] Singh, V., & Johnston, D. (2004). An enzymatic process for corn wet milling. *Advances in Food and Nutrition Research,**48*, 151–171. 10.1016/S1043-4526(04)48003-6 15498695 10.1016/S1043-4526(04)48003-6

[CR120] Somavat, P., Li, Q., Kumar, D., Mejia, E. G. de, Liu, W., Rausch, K. D., Juvik, J. A., Tumbleson, M. E., & Singh, V. (2017). A new lab scale corn dry milling protocol generating commercial sized flaking grits for quick estimation of coproduct yield and composition. *Industrial Crops and Products*. 10.1016/j.indcrop.2017.08.013

[CR121] Somavat, P., Liu, W., & Singh, V. (2021). Wet milling characteristics of corn mutants using modified processes and improving starch yields from high amylose corn. *Food and Bioproducts Processing,**126*, 104–112. 10.1016/j.fbp.2020.12.015

[CR122] Sousa, MFde, Guimarães, R. M., Araújo, Md. O., Barcelos, K. R., Carneiro, N. S., Lima, D. S., Santos, D. C. D., Batista, Kd. A., Fernandes, K. F., Lima, M. C. P. M., & Egea, M. B. (2019). Characterization of corn (*Zea mays* L.) bran as a new food ingredient for snack bars. *LWT,**101*, 812–818. 10.1016/j.lwt.2018.11.088

[CR123] Sukop, U., Hoefler, K., Bender, D., D’Amico, S., Jekle, M., Schoenlechner, R., & Domig, K. J. (2025). Effect of wet fractionation conditions and pulsed electric field on arabinoxylan and protein recovery from maize.* Foods*. 10.3390/foods14050760 10.3390/foods14050760 PMC11898790 40077463

[CR124] Sun, X., Wu, C., Ma, L., & Liang, J. (2025). Positive improvement of nutritional profile and safety of maize processing by-products: Effects of *Bacillus subtilis* fermentation treatment. *LWT,**223*, Article 117775. 10.1016/j.lwt.2025.117775

[CR125] Tabtabaei, S., Kuspangaliyeva, B., Legge, R. L., & Rajabzadeh, A. R. (2023). Air classification of plant proteins. In A. J. Hernández-Álvarez, M. Mondor, & M. G. Nosworthy (Eds.), *Green protein processing technologies from plants* (pp. 31–59). Springer International Publishing. 10.1007/978-3-031-16968-7_2

[CR126] Tadele, D. T., Islam, M. S., & Mekonnen, T. H. (2025). Zein-based nanoparticles and nanofibers: Co-encapsulation, characterization, and application in food and biomedicine. *Trends in Food Science & Technology*. 10.1016/j.tifs.2024.104809

[CR127] Talukder, Z. A., Chhabra, R., Basu, S., Gain, N., Mishra, S. J., Kumar, A., et al. (2025). High amylopectin in waxy maize synergistically affects seed germination and seedling vigour over traditional maize genotypes. *Journal of Applied Genetics*. 10.1007/s13353-024-00877-w 38773055 10.1007/s13353-024-00877-w

[CR128] Wang, J., Suo, G., Wit, M., Boom, R. M., & Schutyser, M. A. (2016). Dietary fibre enrichment from defatted rice bran by dry-fractionation. *Journal of Food Engineering,**186*, 50–57. 10.1016/j.jfoodeng.2016.04.012

[CR129] Wang, J., Tang, J., Ruan, S., Lv, R., Zhou, J., Tian, J., Cheng, H., Xu, E., & Liu, D. (2021). A comprehensive review of cereal germ and its lipids: Chemical composition, multi-objective process and functional application. *Food Chemistry,**362*, Article 130066. 10.1016/j.foodchem.2021.130066 34098434 10.1016/j.foodchem.2021.130066

[CR130] Wang, L., Li, D., Ye, L., Zhi, C., Zhang, T., & Miao, M. (2025). Structure and functionality of micronized maize starch differing in amylose content. *Food Chemistry*. 10.1016/j.foodchem.2025.145534 10.1016/j.foodchem.2025.145534 40684572

[CR131] Wang, S., Wang, J., Yu, J., & Wang, S. (2014). A comparative study of annealing of waxy, normal and high-amylose maize starches: The role of amylose molecules. *Food Chemistry*. 10.1016/j.foodchem.2014.05.055 10.1016/j.foodchem.2014.05.055 24996342

[CR132] Wang, Y., Liu, J., Zhang, Z., Meng, X., Yang, T., Shi, W., He, R., & Ma, H. (2023). Insights into ultrasonication treatment on the characteristics of cereal proteins: Functionality, conformational and physicochemical characteristics. *Foods*. 10.3390/foods12050971 10.3390/foods12050971 PMC10000784 36900488

[CR133] Wen, Y., Niu, M., Zhang, B., Zhao, S., & Xiong, S. (2017). Structural characteristics and functional properties of rice bran dietary fiber modified by enzymatic and enzyme-micronization treatments. *LWT,**75*, 344–351. 10.1016/j.lwt.2016.09.012

[CR134] Wu, S., Peng, Y., Xi, J., Zhao, Q., Xu, D., Jin, Z., & Xu, X. (2022). Effect of sourdough fermented with corn oil and lactic acid bacteria on bread flavor. *LWT,**155*, Article 112935. 10.1016/j.lwt.2021.112935

[CR135] Yada, R. Y. (Ed.). (2018). *Woodhead publishing series in food science, technology and nutrition*. *Proteins in Food Processing (Second Edition)* (Second edition). Woodhead Publishing. https://search.ebscohost.com/login.aspx?direct=true&scope=site&db=nlebk&db=nlabk&AN=1497603

[CR136] Yang, P., Haken, A. E., Niu, Y., Chaney, S. R., Hicks, K. B., Eckhoff, S. R., Tumbleson, M., & Singh, V. (2005). Effect of steeping with sulfite salts and adjunct acids on corn wet-milling yields and starch properties. *Cereal Chemistry*. 10.1094/CC-82-0420

[CR137] Yoshida, T., Tsubaki, S., Teramoto, Y., & Azuma, J. (2010). Optimization of microwave-assisted extraction of carbohydrates from industrial waste of corn starch production using response surface methodology. *Bioresource Technology,**101*(20), 7820–7826. 10.1016/j.biortech.2010.05.011 20542685 10.1016/j.biortech.2010.05.011

[CR138] Yu, T., Jing, S., Jiaxin, L., Aixia, W., Mengzi, N., Xue, G., Lili, W., Liya, L., Fengzhong, W., & Litao, T. (2024). Effects of milling methods on rice flour properties and rice product quality: A review. *Rice Science,**31*(1), 33–46. 10.1016/j.rsci.2023.11.002

[CR139] Yue, Z., Sun, L.-L., Sun, S.-N., Cao, X.-F., Wen, J.-L., & Zhu, M.-Q. (2022). Structure of corn bran hemicelluloses isolated with aqueous ethanol solutions and their potential to produce furfural. *Carbohydrate Polymers,**288*, Article 119420. 10.1016/j.carbpol.2022.119420 35450662 10.1016/j.carbpol.2022.119420

[CR140] Zhang, L., Kitaori, N., & Lai, K. (2025). Process parameters affecting gaba content in functional rice bread. *LWT*. 10.1016/j.lwt.2025.118132

[CR141] Zhang, R., Ma, S., Li, L., Zhang, M., Tian, S., Wang, D., Liu, K., Liu, H., Zhu, W., & Wang, X. (2021). Comprehensive utilization of corn starch processing by-products: A review. *Grain & Oil Science and Technology,**4*(3), 89–107. 10.1016/j.gaost.2021.08.003

[CR142] Zhang, S., Zhang, X., Geng, Z., Liu, X., Wang, Y., Liu, Z., Chen, X., Sun, T., Jin, C., Wang, G., Ji, J., & Liu, H. (2020). Preparation of corn-oil as an alternative fuel and transcriptome analysis of metabolic pathway related to fuel component accumulation. *Fuel,**275*, Article 117931. 10.1016/j.fuel.2020.117931

[CR143] Zhang, Y., Li, F., Huang, K., Li, S., Cao, H., Xie, J., & Guan, X. (2023). Structural changes of starch under different milling degrees affect the cooking and textural properties of rice. *Food Chemistry: X,**17*, Article 100627. 10.1016/j.fochx.2023.100627 36974186 10.1016/j.fochx.2023.100627 PMC10039256

[CR144] Zhao, G., Zhang, R., Dong, L., Huang, F., Tang, X., Wei, Z., & Zhang, M. (2018). Particle size of insoluble dietary fiber from rice bran affects its phenolic profile, bioaccessibility and functional properties. *LWT,**87*, 450–456. 10.1016/j.lwt.2017.09.016

[CR145] Zhao, M., Xiong, W., Chen, B., Zhu, J., & Wang, L. (2020). Enhancing the solubility and foam ability of rice glutelin by heat treatment at pH12: Insight into protein structure. *Food Hydrocolloids,**103*, Article 105626. 10.1016/j.foodhyd.2019.105626

[CR146] Zhao, S., Shi, J., Cai, S., Xiong, T., Cai, F., Li, S., Chen, X., Fan, C., Mei, X., & Sui, Y. (2023). Effects of milling degree on nutritional, sensory, gelatinization and taste quality of different rice varieties. *LWT,**186*, Article 115244. 10.1016/j.lwt.2023.115244

[CR147] Zhiguang, C., Haixia, Z., Min, C., Fayong, G., & Jing, L. (2025). The fine structure of starch: A review. *Npj Science of Food*. 10.1038/s41538-025-00414-x 10.1038/s41538-025-00414-x PMC11985489 40210851

[CR148] Žilić, S., Hadži-Tašković Šukalović, V., Milašinović, M., Ignjatović-Micić, D., Maksimović, M., & Semenčenko, V. (2010). Effect of micronisation on the composition and properties of the flour from white, yellow and red maize. *Food Technology and Biotechnology, 48*(2), 198–206. https://www.ftb.com.hr/images/pdfarticles/2010/April-June/48_198.pdf

