# Supplementary material for: Comparative Study of Fractionation Technologies and Their Impact on the Nutritional and Functional Properties of Maize and Rice Fractions
Source: Food Bioproc Tech. 2025 Dec 6;19(1):33. doi: 10.1007/s11947-025-04104-0 (PMC12681460; doi:10.1007/s11947-025-04104-0)
Supplement: Supplementary file 1 — (DOCX 19.0 KB) [file 11947_2025_4104_MOESM1_ESM.docx]

**Supplementary Table 1** Percentage quantity and nutritional composition of constituents of rice and maize [g/100 g dry matter]

| **Constituents** | **Starch** | **Protein** | **Dietary fibre** | **Fat** |
| --- | --- | --- | --- | --- |
| **Maize (100 % grain)** | 60-68^1^ | 8.5-10.5^1^ | 12-16^1^ | 3.5^1^ |
| Germ (11.1 %^2^) | 8.0^3^ | 18-19^3^ | 14.0^2^ | 20-25^4a^; 40-50^4b^ |
| Bran (5.3 %^2^) | 7.3^2^ | 3.7^2^ | 83.6^2^ | 1.0^2^ |
| Endosperm (82.9 %^2^) | 87.6^2^ | 8.0^2^ | 3.2^2^ | 0.8^2^ |
| **Brown rice (100 % grain)** | 66.4^5^ | 4.3-18.2^5^ | 2.9-4.4^5^ | 1.6-2.8^5^ |
| Germ (4 %^2^) | 2.4^2^ | 20.2^2^ | 3.5^2^ | 21.6^2^ |
| Bran (3 %^2^) | 16.1-26.7^6^ | 11.3-14.9^4^ | 19-29^4^ | 15.0-19.7^4^ |
| Endosperm (93 %^2^) | 90.2^2^ | 7.8^2^ | 0.4^2^ | 0.5^2^ |

^1^(Serna-Saldivar 2023); ^2^(Kulp & Ponte, 2000); ^3^(Deepak und Jayadeep 2022); ^4^(Zheng et al. 2018), ^a^from dry-milling, ^b^from wet-milling; ^5^ (Pereira et al. 2021); ^6^(Farooq und Yu 2025)

**Supplementary Table 2** Osborne fractions [%] of whole kernels and constituents of maize and rice

|  | **Rice** (Cao et al., 2009) | | | **Maize** | | | |
| --- | --- | --- | --- | --- | --- | --- | --- |
|  | **Whole kernel** | **Endosperm** | **Bran** | | **Whole kernel^1^**  (Bolotova et al., 2023) | **Endosperm^2^**  (Choudhary & Chaudhary, 2020) | **Germ (embryo)**  (Parris et al., 2006) |
| **Albumin** | 9.4 | 6.2 | 42.7 | | 14.22 | 1.4-7.9 | 34.1^3^; 22.6^4^ |
| **Globulin** | 7.5 | 6.0 | 12.5 | | 9.12 | 1.3-9.4 | 28.0^3^; 2.3^4^ |
| **Prolamin**^5^ | 5.5 | 6.9 | 3.2 | | 19.5 | 20.2-47.8 | 4.6^3^; 0.9^4^ |
| **Glutelin**^6^ | 75.0 | 78.8 | 40.3 | | 23.7 | 16.2-25.2 | 33.3^3^; 74.2^4^ |

^1^Average of 12 varieties; ^2^six varieties; ^3^obtained via dry-fractionation;, ^4^wet-fractionation; ^5^oryzin in rice, zein in maize; ^6^oryzenin in rice, zeanin in maize

**References**

Kulp, K., & Ponte, J. G. JR (Eds.). (2000). Handbook of cereal science and technology (2nd ed.). CRC Press.

Zheng, L., Ji, C., Jin, J., Xie, D., Liu, R., Wang, X., et al. (2018). Effect of moisture and heat treatment of corn germ on oil quality. Journal of the American Oil Chemists’ Society, 95(3), 383–390. https://doi.org/10.1002/aocs.12032

Bolotova, O. I., Sazonova, I. A., & Bychkova, V. V. (2023). Fractional composition of protein polymers in corn grain. Russian Agricultural Sciences, 49(3), 271–275. https://doi.org/10.3103/S1068367423030047

Choudhary, P., & Chaudhary, D. P. (2020). Comparison of protein composition of normal and quality protein maize. InternationalJournal of Current Microbiology and Applied Sciences, 9(12), 3297–3302. <https://doi.org/10.20546/ijcmas.2020.912.392>

Parris, N., Moreau, R. A., Johnston, D. B., Singh, V., & Dickey, L. C. (2006). Protein distribution in commercial wet- and drymilled corn germ. Journal of Agricultural and Food Chemistry, 54(13), 4868–4872. https://doi.org/10.1021/jf060336d . Vijay.
